# Functional plasticity shapes neutrophil response to *Leishmania major* infection in susceptible and resistant strains of mice

**DOI:** 10.1371/journal.ppat.1012592

**Published:** 2024-10-08

**Authors:** Thiago DeSouza-Vieira, Marco Antônio M. Pretti, Phillipe Souza Lima Gomes, Heitor A. Paula-Neto, Amy Goundry, Michelle T. Nascimento, Sundar Ganesan, Triciana Gonçalves da Silva, Olena Kamenyeva, Juraj Kabat, Javier Manzella-Lapeira, Fábio B. Canto, Vanderlei da Silva Fraga-Junior, Mateus Eustáquio Lopes, Leonardo Gomes Vaz, Gabriela Pessenda, Andrea Paun, Anita L. Freitas-Mesquita, José Roberto Meyer-Fernandes, Mariana Boroni, Maria Bellio, Gustavo Batista Menezes, Joseph Brzostowski, Jeremy Mottram, David Sacks, Ana Paula C. A. Lima, Elvira M. Saraiva

**Affiliations:** 1 Laboratório de Imunobiologia das Leishmanioses, Departamento de Imunologia, Instituto de Microbiologia Paulo de Góes, Universidade Federal do Rio de Janeiro, Rio de Janeiro, Brasil; 2 Laboratório de Bioinformática e Biologia Computacional, Divisão de Pesquisa Experimental Translacional, Instituto Nacional do Câncer (INCA), Rio de Janeiro, Brasil; 3 Laboratório de Alvos Moleculares, Departamento de Biotecnologia Farmacêutica, Faculdade de Farmácia, Universidade Federal do Rio de Janeiro, Rio de Janeiro, Brasil; 4 Laboratório de Bioquímica e Biologia Molecular de Proteases, Instituto de Biofísica Carlos Chagas Filho, Universidade Federal do Rio de Janeiro, Rio de Janeiro, Brasil; 5 Biological Imaging Section, Research Technologies Branch, National Institute of Allergy and Infectious Diseases, National Institutes of Health, Bethesda, Maryland, United States of America; 6 National Center for Structural Biology and Bioimaging, CENABIO, Universidade Federal do Rio de Janeiro, Brazil; 7 Laboratory of Immunogenetics, National Institute of Allergy and Infectious Diseases, National Institutes of Health, Rockville, Maryland, United States of America; 8 Laboratório de Tolerância Imunológica e Homeostase Linfocitária, Departamento de Imunobiologia, Universidade Federal Fluminense, Rio de Janeiro, Rio de Janeiro, Brasil; 9 Laboratório de Imunologia Molecular e Celular, Instituto de Biofísica Carlos Chagas Filho, Universidade Federal do Rio de Janeiro, Rio de Janeiro, Brasil; 10 Centro de Biologia Gastrointestinal, Departamento de Morfologia, Instituto de Ciências Biológicas, Universidade Federal de Minas Gerais, Belo Horizonte, Minas Gerais, Brasil; 11 Departamento de Bioquímica e Imunologia, Instituto de Ciências Biológicas, Universidade Federal de Minas Gerais, Pampulha, Belo Horizonte, Minas Gerais, Brasil; 12 Laboratory of Parasitic Diseases, National Institute of Allergy and Infectious Diseases, National Institutes of Health, Bethesda, Maryland, United States of America; 13 Instituto de Bioquímica Médica Leopoldo de Meis, Centro de Ciências da Saúde, Universidade Federal do Rio de Janeiro, Rio de Janeiro, Brasil; 14 Laboratório de Imunobiologia, Departamento de Imunologia, Instituto de Microbiologia Paulo de Góes, Universidade Federal do Rio de Janeiro, Rio de Janeiro, Brasil; 15 York Biomedical Research Institute and Department of Biology, University of York, York, United Kingdom; University of Geneva Faculty of Medicine: Universite de Geneve Faculte de Medecine, SWITZERLAND

## Abstract

Neutrophils rapidly infiltrate sites of infection and possess several microbicidal strategies, such as neutrophil extracellular traps release and phagocytosis. Enhanced neutrophil infiltration is associated with higher susceptibility to *Leishmania* infection, but neutrophil effector response contribution to this phenotype is uncertain. Here, we show that neutrophils from susceptible BALB/c mice (B/c) produce more NETs in response to *Leishmania major* than those from resistant C57BL/6 mice (B6), which are more phagocytic. The absence of neutrophil elastase contributes to phagocytosis regulation. Microarray analysis shows enrichment of genes involved in NET formation (mpo, pi3kcg, il1b) in B/c, while B6 shows upregulation of genes involved in phagocytosis and cell death (Arhgap12, casp9, mlkl, FasL). scRNA-seq in *L*. *major*-infected B6 showed heterogeneity in the pool of intralesional neutrophils, and we identified the N1 subset as the putative subpopulation involved with phagocytosis. In vivo, imaging validates NET formation in infected B/c ears where NETing neutrophils were mainly uninfected cells. NET digestion in vivo augmented parasite lymphatic drainage. Hence, a balance between NET formation and phagocytosis in neutrophils may contribute to the divergent phenotype observed in these mice.

## Author summary

Leishmaniasis is a complex of vector-borne diseases transmitted by infected sandflies carrying *Leishmania*. Clinical manifestations range from cutaneous lesions to visceral disease, which can be fatal if not treated. Cutaneous Leishmaniasis is the most common clinical manifestation. Experimental models built in the early 70s have established susceptible (BALB/c) and resistant (C57BL/6) strains to *Leishmania major* infection. Neutrophils comprise the first line of defense against microbial invaders, and possess powerful microbicidal tools, which can thwart most pathogens. Accordingly, genetic disorders that lead to neutropenia enhance patients’ susceptibility to infections. Counterintuitively, neutrophilia is associated with enhanced susceptibility to cutaneous Leishmaniasis. Although these cells used to be considered transcriptionally rudimental, currently, neutrophils have emerged as functionally plastic cells. Here, we deepened the understanding of why neutrophils are associated with susceptibility to infection in the murine model by showing that not just the influx, but the functional profile of these cells likely impacts the early response to infection and affects parasite retention in the primary site of infection, which likely contributes to shaping disease outcome. Our results provide mechanistic insights to assist in the development of target-based therapies.

## Introduction

The *Leishmania* genus comprises a diverse group of 20 species of protozoan parasites that cause Leishmaniasis, a complex vector-borne disease whose clinical manifestations range from self-healing skin lesions to the visceral and most lethal form. Transmission to mammalian hosts occurs by the bite of hematophagous phlebotomine sandflies, lacerating capillaries, and regurgitating infective metacyclic forms in host skin [1].

Cutaneous Leishmaniasis (CL) is the most common clinical manifestation, affecting 0.7 to 1 million people annually across 98 countries. CL is characterized by ulcerative skin lesions that could lead to serious disability, life-long scars, and social stigma [1]. *Leishmania major* is the causative agent of CL in the Old World, while *L*. *amazonensis* and *L*. *braziliensis* cause CL and mucocutaneous leishmaniasis in the New World. In animal models, the severity of *Leishmania major* infection has been associated to T cell polarization patterns observed in certain host genetic backgrounds, e.g., BALB/c (B/c), susceptible mice, which preferentially mount type 2 immunity. In contrast, resistant mice, C57BL/6 (B6), mount a Th1 response [2]. Accordingly, parasite growth is significantly higher in mice deficient in IL-12-p40, IFN-γ, or iNOS [3]. However, the disruption of STAT6 leads to the polarization of Th1 and the establishment of early infection by attracting permissive monocytes [4]. Thus, despite the protective effect of Th1 immunity, this mechanism is insufficient to control parasite amplification, and other components and cell types of the immune system must be required to control parasitism throughout the course of the disease. In fact, neutrophils from resistant mice upregulate expression of TLR2, TLR7, and TLR9 and produce IL-10 and biologically active IL-12p70 in response to *L*. *major* invasion, while neutrophils from susceptible mice produce inhibitory IL-12p40 homodimers and upregulate CD49d instead, indicating distinct phenotypes that may influence disease outcome as well [5].

Neutrophils are the first-line responders, rapidly mobilized to infection sites to amplify inflammation initiated by tissue-resident cells [6]. Although formerly branded as short-lived suicide effector cells that promote deregulated inflammation and tissue damage in response to infection, it is currently well-known that neutrophils are complex cells that can switch phenotypes based on endogenous cues they sense in the tissue microenvironment and microbial products. This ability to switch phenotypes has significant implications for cancer immunity [7], sepsis [8], autoimmunity [9], and inflammatory diseases [10].

Accordingly, neutrophils are the first phagocytes recruited to sand fly bite sites to respond to the infective inoculum [6]. Neutrophils can shape the early immune response against *Leishmania sp*. through IL-1β secretion [11], elastase [12–14], and apoptosis [15], which, respectively, modulate parasite dissemination, macrophage response and antigen presentation by dendritic cells, and CD8^+^ T cell priming. Moreover, regulation of neutrophilic IL-1β secretion by tissue-resident macrophages producing heme oxygenase-1 mediates host tolerance to disease and prevents excessive tissue damage [11,16]. Altogether, those findings attest to the importance of neutrophils in shaping host defense against infection.

NETs are web-like structures with a chromatin backbone decorated with cytoplasmic and granular proteins produced by a fraction of the neutrophil pool as a defense mechanism against microbial invasion but also induced by signals produced during sterile inflammation [17]. Human neutrophils release NETs in response to *Leishmania* promastigotes, and *L*. *amazonensis* is susceptible to histone-mediated killing [18]. However, certain species, such as *L*. *donovani* and *L*. *infantum* can evade NET killing [19,20]. Furthermore, *Lu*. *longipalpis* (sandfly) saliva also possesses a nuclease activity that causes NET degradation [21]. However, the biological significance of neutrophil extracellular traps to the course of *Leishmania* infection remains to be addressed.

Neutrophil phagocytosis of metacyclic promastigotes has been proposed to shield parasites inside neutrophils, acting as “Trojan horses” protecting them until transfer to macrophages, their permanent host cells, by efferocytosis or by direct parasite transfer [22,23]. However, it has been shown that neutrophils may also allow parasite replication [24] and can kill some *Leishmania* species [25]. Interestingly, evidence suggests that NET formation and phagocytosis are divergent pathways initiated by a distinct set of neutrophil subpopulations [26–28].

In the present study, we provide evidence for a dichotomy in neutrophil microbicidal response in B/c and B6 mice, supported by plasticity in the expression of genes related to phagocytosis and NET formation in the skin transcriptome. We found transcriptional heterogeneity in the pool of neutrophils infiltrating the infected tissue, which may also contribute to shaping the phenotype of these cells. Finally, we correlate NET formation at the site of infection to the control of parasite lymphatic drainage. Hence, we propose that plasticity in the pool of neutrophils from each mouse strain may influence host susceptibility to infection.

## Results

### BALB/c (B/c) neutrophils are more efficient at NET production than C57BL/6 (B6) and differ in neutrophil elastase content

**Fig 1:**
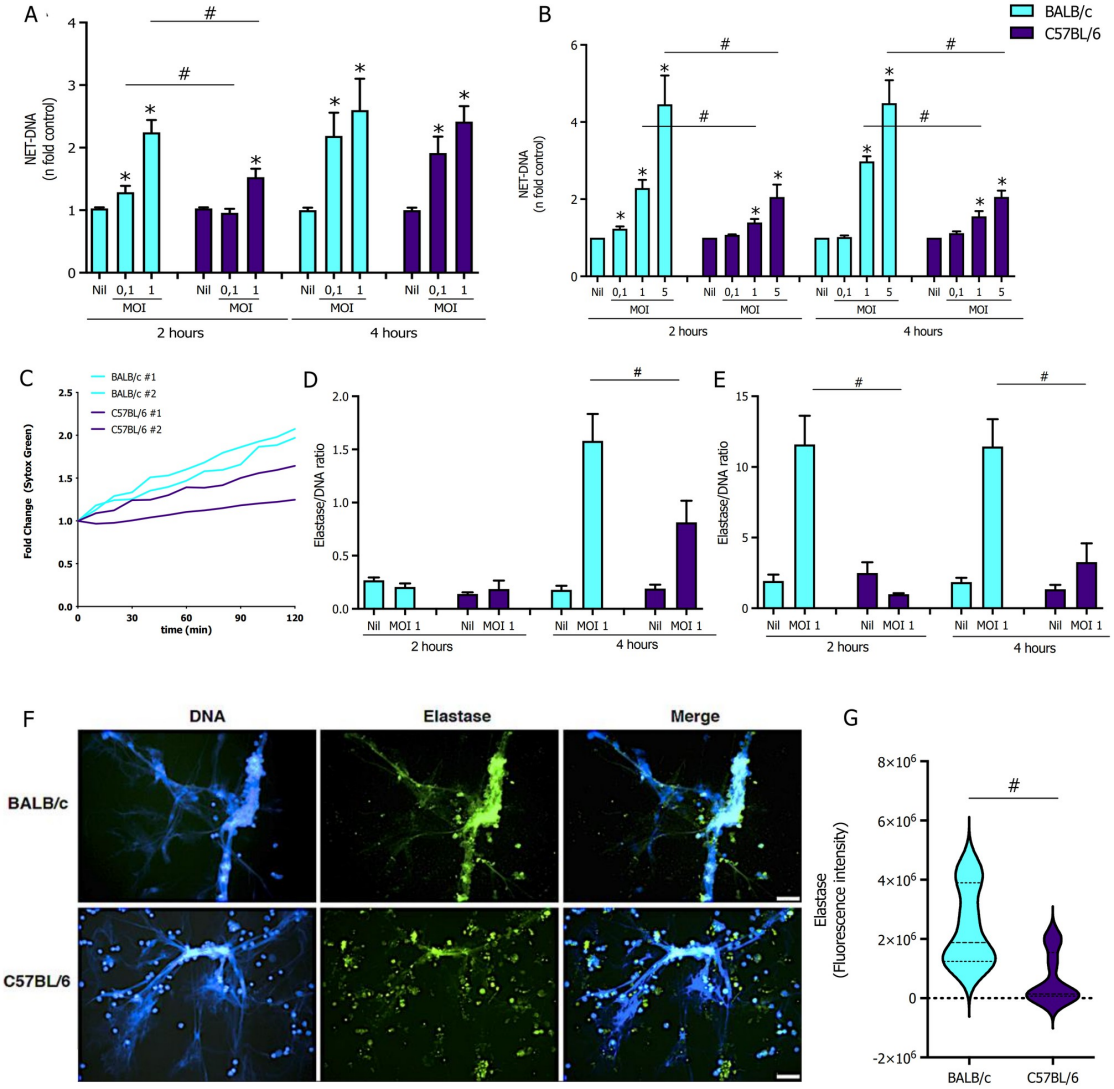
Neutrophils from B/c mice show greater potential to generate NETs enriched in elastase than B6 mice. Bone marrow neutrophils (BMN; 5 x 10^5^ cells/ well) of B/c (blue bars) or B6 (purple bars) mice were either left untreated (Nil) or incubated with the increasing MOIs indicated of *L*. *major* promastigotes for 2 or 4 h. We quantified **(A)** NETs as extracellular dsDNA from BMN (1 x 10^6^ cells/ well), which was represented as a fold increase over control. **(B)** Casein-recruited inflammatory neutrophils (_i_NØs; 1 x 10^6^ cells/ well) of B/c or B6 mice were incubated with an increasing ratio of *L*. *major* promastigotes for 2 or 4 h and we quantified NETs and followed **(C)** kinetics of NET formation by Sytox Green staining every 10 min for a period of 120 min. **(D)** Quantifying NETs composition by elastase/dsDNA ratio of BMN and **(E)**_i_NØs. **(F)** Immunofluorescence of NETs stained with Hoechst (blue) and anti-neutrophil elastase (green). **(G)** Quantification of mean elastase fluorescence intensity from 2 independent experiments performed in duplicate. Scale bar: 20 μm. Results are mean±SEM of n = 4–10 performed as 2–3 independent experiments. Differences (*) relative to Nil or (#) between indicated bars were considered significant when p<0.05.

Experiments in this section were conducted to determine the capacity of neutrophils from B/c and B6 mice to produce NETs in response to *L*. *major* infection. Neutrophils were isolated from bone marrow (BMN) and were incubated with an increasing ratio of *L*. *major* promastigotes. NETs were quantified by measuring supernatants’ free dsDNA and neutrophil elastase (NE) activity. NET-DNA production was normalized based on basal levels of dsDNA produced by quiescent cells from each experiment performed side-by-side (S1A Fig). Fig 1A shows that B/c BMN outperformed B6 BMN in NET-DNA production 2h post-stimulation (p.s.). Only the highest parasite ratio elicited a response from B6 BMN during this period. In contrast, 4 h p.s. NET-DNA levels were equivalent among mouse strains (Fig 1A). Additionally, after parasite stimulation, extracellular levels of NE activity increased in a parasite-dependent manner in B/c BMN but remained stable in B6 BMN (S1B Fig).

To address if this response pattern would persist under inflammation, we collected inflammatory neutrophils (_i_NØ) elicited to the peritoneal cavity by casein hydrolysate i.p. injection. Similarly, NET-DNA released by B/c _i_NØ was significantly higher than B6 at 2- and 4 h p.s. (Fig 1B). Kinetics of NET extrusion measured with Sytox Green vital dye every 10 min for 2 h confirmed the reduced capacity of B6 iNØ to produce NETs in comparison to B/c upon contact with promastigotes (Fig 1C). Moreover, injection of metacyclic promastigotes into the peritoneal cavity led to significantly higher levels of NET-DNA in the lavage fluids collected from B/c mice (S2A Fig). However, neutrophils were not the major population in the inflammatory infiltrate elicited by metacyclic promastigotes in the peritoneal cavity (S2B Fig). Yet, the total numbers of neutrophils recruited from each mouse strain were equivalent (S2C Fig).

Distinct extracellular NE activity observed with the *Leishmania* stimulated neutrophils in vitro suggests that NETs from B/c and B6 neutrophils may differ in NE content. Therefore, we estimated the relative levels of elastase associated with the DNA backbone of NETs by calculating the ratio of extracellular elastase activity per dsDNA arbitrary units. The elastase/DNA ratios were equivalent in the B/c BMN and B6 BMN supernatants at 2 h p.s. In contrast, we detected a ~50% increase in the elastase/DNA ratio of B/c compared to B6 at 4 h p.s. (Fig 1D). Furthermore, B/c _i_NØ elastase/DNA ratio was 10-fold and 5-fold higher than B6 _i_NØ after 2 and 4 h of stimulation with promastigotes, respectively (Fig 1E). Finally, immunofluorescence confirmed differences between B/c and B6 in the levels of elastase decorating NET-DNA microfibers, which was further supported by quantification of pixel intensity of NE staining co-localized with DNA microfibers (Fig 1F and 1G).

Reproducibility of studies performed in mice in a single laboratory can be challenging due to discrepancies in animal husbandry, which can affect the microbiome and behavior of these animals and, as a result, reshape the immune system. To certify the consistency of our findings, we used B/c and B6 strains acquired from Taconic Biosciences and housed at another animal facility. Additionally, experiments were conducted following a distinct experimental protocol to avoid bias. Briefly, highly purified BMN obtained by magnetic negative selection was stimulated with purified metacyclic promastigotes for 3 h, and extracellular dsDNA was measured in a NanoDrop. Accordingly, we observed the same strain differences in NET-DNA production (S3 Fig). Hence, we demonstrate that B/c BMN responds differently to *L*. *major* infection regarding NET formation when compared to B6 neutrophils, and, importantly, this phenotype was intensified by inflammatory priming.

### *Leishmania major*-induced NET formation in BMN requires neutrophil elastase but is independent of NADPH oxidase and TLR4

**Fig 2:**
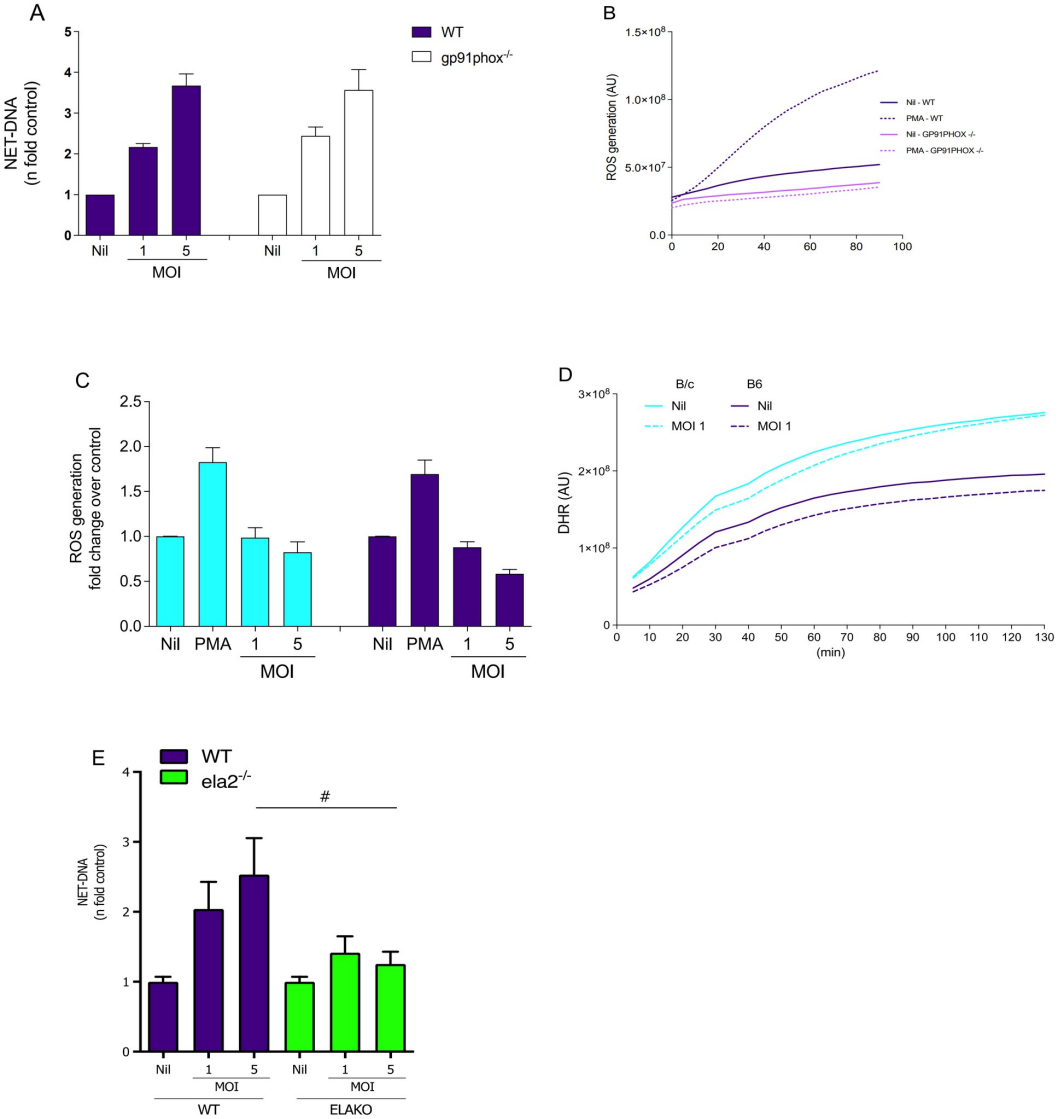
NET induction by *Leishmania major* is independent of NADPH oxidase activity. (A) BMN (5 x 10^5^ cells/ well) of B6 wild-type (WT; gray bars) or *gp91phox*^*-/-*^ (hatched bars) mice were either left untreated (Nil) or incubated with increasing numbers of *L*. *major* promastigotes (1:1, 1:5) and NETs measured as the release of extracellular dsDNA and presented as fold increase over control 4 h post-stimulation. (B) ROS generation of bone-marrow-derived neutrophils of wild-type (black lines) or *gp91phox-/-* (purple lines) mice were measured every 5 min for a period of 90 min in the presence (filled lines) or absence of PMA (dashed lines). (C) BMN (5 x 10^5^ cells/ well) of B/c (blue bars) or B6 (purple bars) mice were either left untreated (Nil) or incubated with an increasing ratio of *L*. *major* promastigotes (MOI 1 and 5) or PMA (100 nM) and (D) ROS generation was measured during 2 h with DHR123 probe. (E) Quantification of NET-DNA release 1 h post-stimulation with *L*. *major* promastigotes (MOI 1 and 5) of BMN from wild type (purple bars) and elastase knockout (green bars) mice. Results are mean±SEM of n = 4–6. Difference (#) between indicated bars were considered significant when p<0.05.

To investigate the involvement of ROS produced by NADPH oxidase in NET release, B6 BMN from *gp91phox*^-/-^ or wild-type mice were stimulated with *L*. *major* promastigotes for 4 h, and we detected equivalent levels of NET-DNA extrusion in neutrophils from both strains (Fig 2A). To confirm the absence of the NADPH oxidase complex activity on *gp91phox*^*-/-*^, we evaluated ROS generation induced by PMA on *gp91phox*^*-/-*^ or B6 wild-type BMN. As expected, ROS generation was not detected in knockout mice, which is in contrast to the high levels of ROS produced by wild-type BMN (Fig 2B). We evaluated the capacity of BMN from B/c and B6 to produce ROS in response to *L*. *major* promastigotes. BMN from both strains showed equivalent ROS production in response to PMA (Fig 2C). Interestingly, promastigotes did not induce detectable changes in the levels of ROS on BMN from either strain (Fig 2D).

To address the role of elastase on NETs induced by *L*. *major*, we isolated B6 BMN from elastase knockout (ela2^-/-^) or B6 wild-type mice and stimulated them with promastigotes. We observed that ela2^-/-^ mice failed to form NETs beyond quiescent levels, confirming the role of NE expression on NET formation (Fig 2E).

Additionally, since TLR4 participates in the *Leishmania* sp. recognition [29,30], we tested the participation of this receptor in the NET formation induced by the parasite. Our results showed that TLR4 absence did not affect NET formation induced by *L*. *major* in total bone marrow cells (S4 Fig). Thus, we demonstrated that induction of NETs by *L*. *major* does not require assembling the NADPH oxidase complex or TLR4 but is dependent on neutrophil elastase-mediated chromatin decondensation.

### Elastase as a key checkpoint regulator of neutrophil effector response

**Fig 3:**
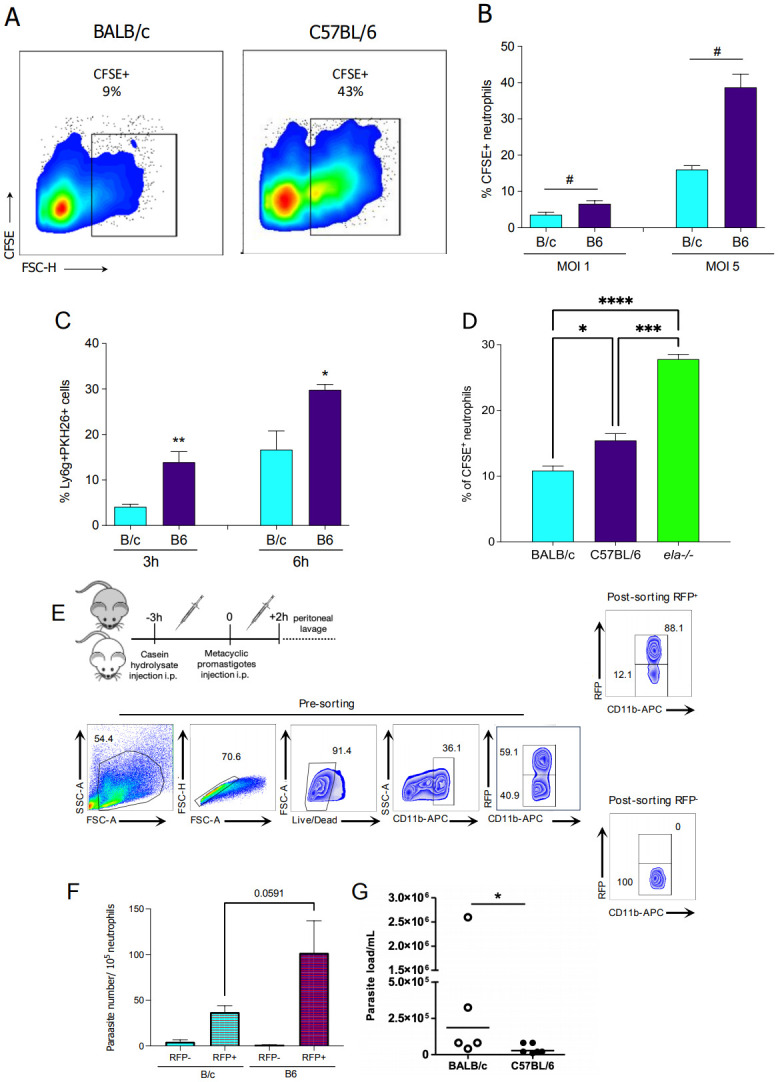
B6 neutrophils are more effective in phagocytosis than B/c, but promastigotes can evade killing. (A) Representative scatter plots of the percentage of _BMN_CFSE^+^ from B/c and B6 mice incubated with CFSE-labeled parasites and (B) data compilation. (C) Percentage of PKH26^+^ cells among recruited neutrophils from mouse ears 3 h after injection of PKH26-labeled parasites. (D) Percentage of _BMN_CFSE^+^ from B/c, B6, and ELA^-/-^ mice incubated with CFSE-labeled parasites. (E) _i_NØ recovered from peritoneal lavage fluid 3h post-injection of casein hydrolysate and 2h after intraperitoneal injection with 2×10^6^ *L*. *major*-RFP stationary phase promastigotes were sorted to obtain uninfected RFP^-^ and infected RFP^+^_i_NØ from B/c and B6 mice. Representative dot plots of gated cells display quadrant values as a percentage of each gate during pre-sort and post-sorting. (F) Parasite load recovered from uninfected RFP^-^ and infected RFP^+^_i_NØ from B/c and B6 mice calculated by limiting dilution assay. (G) Parasite load in the skin 18 h post-infection from B/c (white dots) and B6 (black dots) mice. Results are compilation of 2–3 independent experiments. Results are mean±SEM of n = 4–6. Differences (*) relative to Nil or (#) between indicated bars were considered significant when *p<0.05.

At increasing ratios, we assessed neutrophils’ capacity to uptake parasites by incubating CFSE-labeled promastigotes with B6 or B/c BMN. SSC^high^Ly6G^+^ cells from B6 mice showed, on average 2-fold higher percentage of CFSE^+^ cells than B/c BMN (Fig 3A and 3B). To further explore this phenomenon *in vivo*, we injected PKH26-labeled metacyclic promastigotes intradermally into mouse ears. Fluorescence-activated cell sorting analysis on dermal cells at 3 and 6h post-injection showed a significant increase (3- and 2-fold, respectively) in the percentage of PKH26^+^Ly6G^+^ infected neutrophils from B6, compared to B/c mice (Fig 3C). Next, we observed that NE-deficiency leads to increased neutrophil phagocytosis of *L*. *major*-CFSE^+^ parasites *in vitro* compared to wild-type mice, indicating that NE is a key checkpoint regulator of neutrophil effector response since in its absence, phagocytosis takes precedence over NET formation in the neutrophil response (Fig 3D). To evaluate intracellular parasite viability in neutrophils, we elicited iNØ and subsequently infected the peritoneal cavity with *Leishmania major*-RFP parasites. We sorted uninfected RFP- and infected RFP+ neutrophils as CD11b^high^SSC-A^high^, as depicted in the pre-sorting gating strategy, and we further confirmed sorting efficiency by post-sorting analysis of samples (Fig 3E). We recovered viable parasites in neutrophils from both mice, whilst B6 neutrophil displayed a 2-fold increase in parasite numbers, which reached borderline statistical significance, p = 0.0591, (Fig 3F). Hence, despite differences in phagocytic capacity, neutrophils from both mice retain viable promastigotes inside them.

Finally, to compare the level of parasite viability in the skin of these mice during the onset of inflammation at the site of infection, we injected i.d. B/c and B6 mice with 10^6^ metacyclic promastigotes of *L*. *major* and measured parasite load in the ear skin 18 h post-injection, since at this stage parasites have not gone through replication and neutrophils remain the majority of the inflammatory infiltrate. B6 mice exhibited a diminished number of parasites in the ears compared to B/c, indicating that early inflammatory response in B6 mice is more effective at killing parasites, which reduces the number of parasites available to establish infection (Fig 3G).

**Fig 4:**
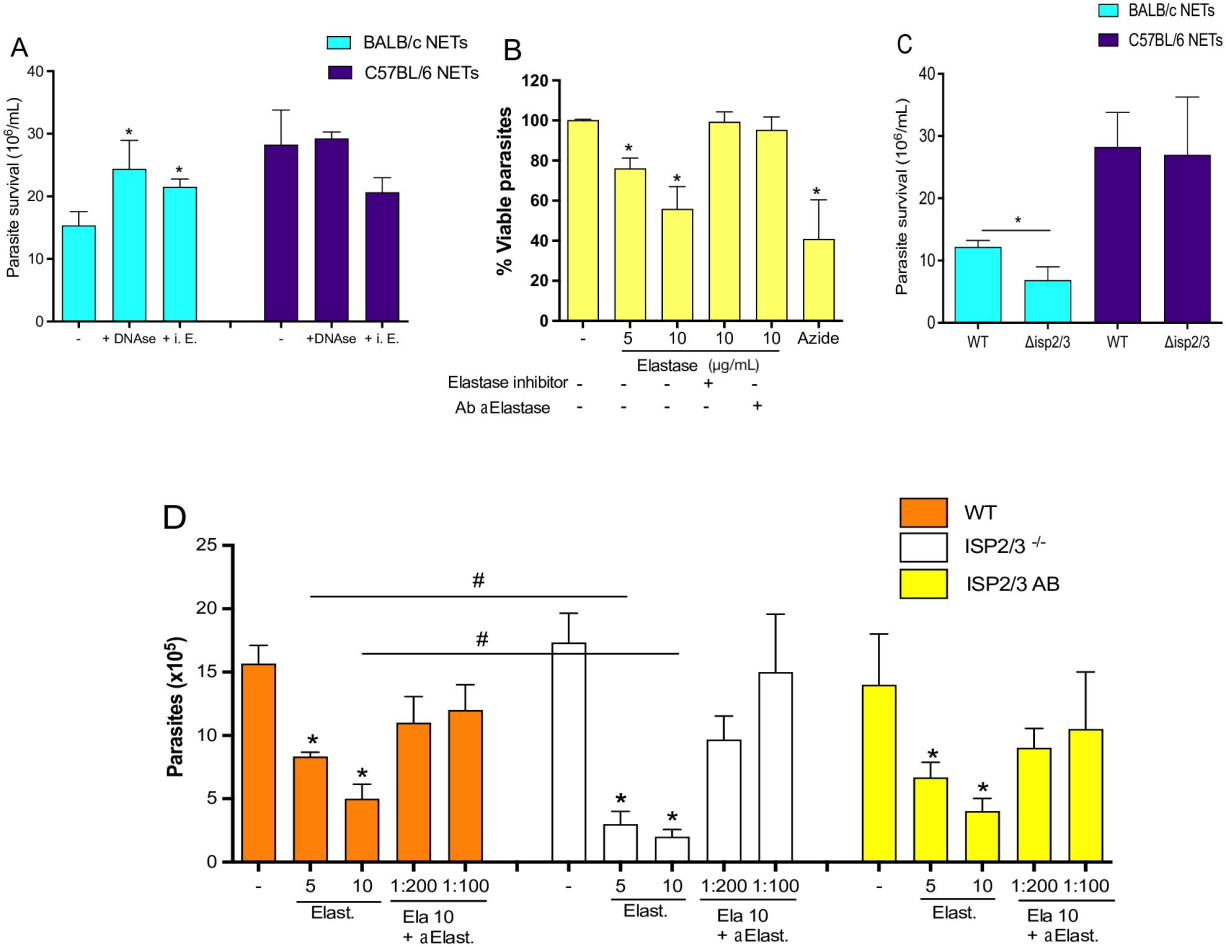
ISP2/3 mediates evasion to NET killing promoted by neutrophil elastase. (A) _i_NØs were stimulated with FA-fixed promastigotes, and NET-enriched supernatant was incubated with live promastigotes in the presence of 100 units/mL DNase or 20 μg/mL elastase inhibitor (i.E) to determine NET-mediated parasite killing measured as parasite survival. Purified recombinant elastase was incubated with promastigotes alone or in combination with either Ab anti-elastase (1:100) or elastase inhibitor 20 μg/mL, and (B) percentage of viable parasites was measured by XTT Cell Viability Assay. (C) NET-killing assay comparing WT or Δisp2/3 parasite strains. (D) The number of viable WT, Δisp2/3, and Δisp2/3 add back (Δisp2/3 AB) parasites incubated with elastase (5 μg/mL and 10 μg/mL; Ela 10) and the indicated dilutions of anti-elastase antibody. Results are mean±SEM of n = 4–6. Differences (*) relative to Nil or (#) between indicated bars were considered significant when p<0.05.

Next, to explore the toxicity of NETs to promastigotes, we generated NET-enriched supernatants from _i_NØ obtained from each mouse strain, and incubated them with *L*. *major* overnight at 35°C, human skin temperature. Additionally, supernatants were pre-treated with DNAse or elastase inhibitor. We observed that NETs derived from B/c mice killed 50% more promastigotes than B6 mice-derived NETs (Fig 4A). DNAse treatment and elastase inhibition significantly increased parasite survival when co-incubated with B/c-derived NETs. In contrast, treatments with DNAse or elastase inhibitors did not affect parasite viability in cultures treated with B6-derived NETs (Fig 4A). Moreover, incubation of promastigotes with purified human leukocyte elastase (HLE) promoted parasite killing in a dose-dependent manner (Fig 4B). Accordingly, promastigote viability was rescued by elastase inhibition or by anti-elastase neutralizing antibodies (Fig 4B). Moreover, we tested whether HLE was up taken by *L*. *major* by incubating promastigotes with HLE for 2 h, and subsequently, stained parasites intracellularly for elastase. We observed staining for HLE at the cell surface, flagellum, and cytosol (S5 Fig), which indicates that HLE interacts with the parasite membranes reaching the cytosol, where it can provoke killing through yet unknown mechanisms. It was previously shown that a parasite protein that inhibits neutrophil elastase, ISP2, inhibits the proteolytic activity of NE at the host cell, with consequences for macrophage infection [31]. Hence, we sought to evaluate the role of parasite ISPs on NETs killing evasion. Firstly, *L*. *major* mutants deficient in ISP2 and ISP3 (Δ*isp2/3*) or wild-type strains were treated with NET-enriched supernatants. Accordingly, we observed similar parasite survival when Δ*isp2/3* or WT promastigotes were treated with NETs from B6 mice (Fig 4C). In contrast, Δ*isp2/3* promastigotes were significantly more susceptible to killing mediated by B/c NETs compared to wild-type parasites (Fig 4C). Accordingly, purified elastase was significantly more toxic to Δ*isp2/3* parasites compared to wild-type or add-back strains and, this phenotype was prevented by NE neutralizing antibodies (Fig 4D). Therefore, we demonstrate that, although elastase promotes some level of parasite killing, promastigotes can evade NET killing through the expression of ISP2 and persist inside infected neutrophils of both mice.

### Myeloperoxidase (MPO) also participates in *Leishmania major*-induced NET formation

Since MPO is also associated with the NET scaffold and is involved in chromatin decondensation, we analyzed the presence of this enzyme in casein-stimulated neutrophils from both mouse strains. Our findings demonstrated that MPO is associated with the NET-DNA scaffold from both mouse strains. (S7 Fig).

To further address differences in the MPO mobilization in response to global activation, we evaluated the subcellular distribution of MPO in the cytoplasmic and nuclear fractions of BMN stimulated with PMA/ionomycin, a pan-activator cocktail. Cytoplasmic MPO was significantly depleted in neutrophils from B/c compared to B6 mice 15 and 30 min p.s., while levels in both strains were similarly restored by 1h p.s. (S8A Fig). The amount of intranuclear MPO at steady-state was significantly higher in B/c BMN compared to B6 (S8B Fig). Interestingly, we also observed a transient depletion of MPO staining in the nucleus 15 and 30 min p.s. Moreover, 1 h p.s. the intensity of intranuclear MPO was significantly greater in B/c BMN compared to neutrophils from B6 BMN, which demonstrates that neutrophils from B/c preferably mobilize MPO into the nucleus in response to activation (S8B Fig). 3D reconstruction confirms subcellular localization of MPO inside the nucleus of BMN (S1 Video). We did not detect NET-DNA release at this activation point (S8C Fig). Likewise, higher staining of MPO in the nucleus of B/c neutrophils at steady-state and enhanced mobilization of MPO to the nucleus upon pan-activation suggests that B/c mice are more prone to respond to *L*. *major* infection by releasing NETs than neutrophils from B6 mice.

### Comprehensive transcriptional analysis supports the plasticity of neutrophil effector response at the host and cellular levels

**Fig 5:**
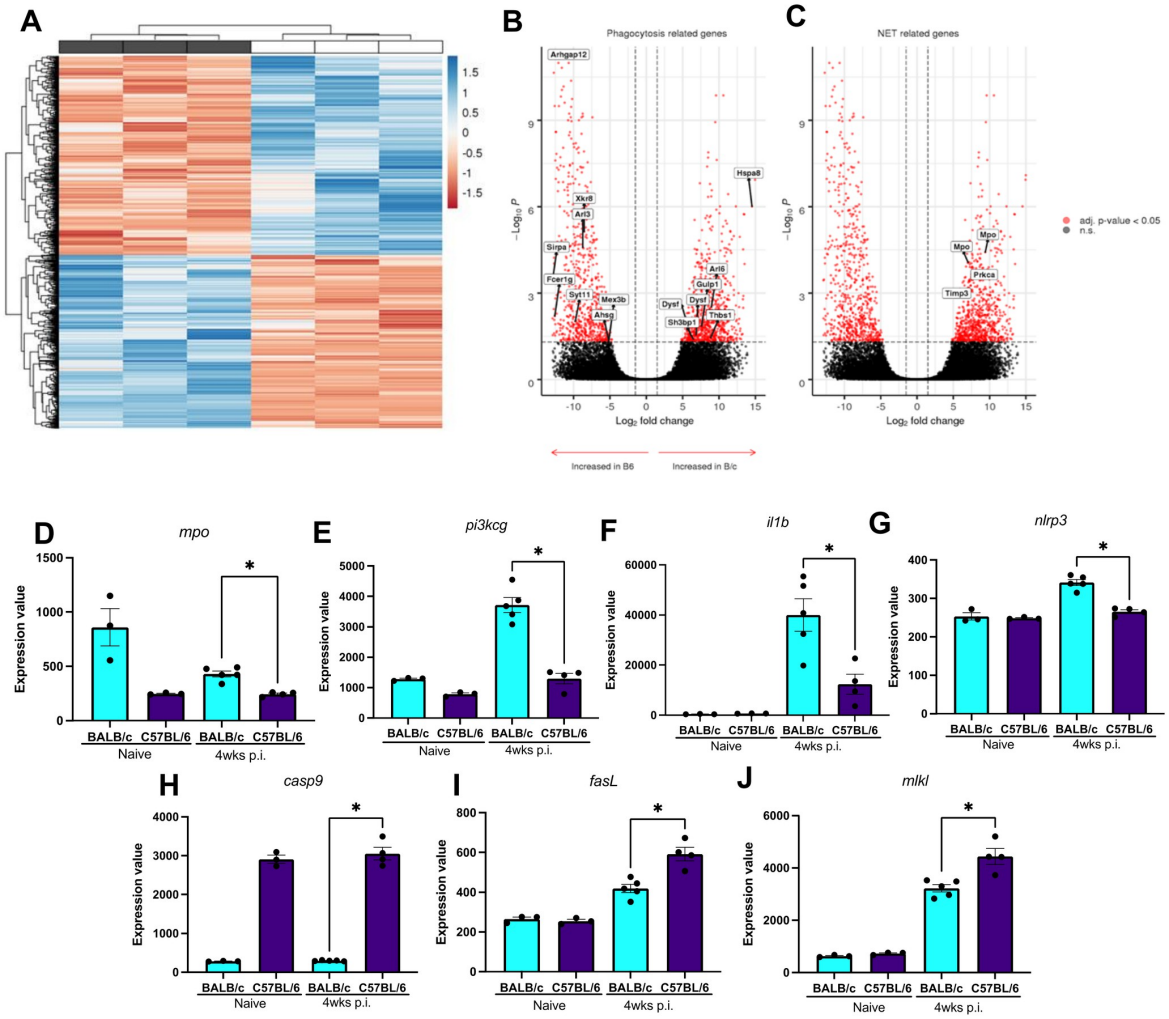
Transcriptional profiling of genes related to NET release and different types of cell death and phagocytosis in C57BL/6 (B6) versus BALB/c (B/c). (A) Processed transcriptome array of B/c and B6 naïve mice was analyzed to identify differentially expressed probes. 1,489 differentially expressed probes were detected using the LIMMA package, of which 795 were increased in the B/c naive and 694 in the B6 naive condition. Significant probes were selected using a threshold of adjusted p-value < 0.05 and log2 fold change > 1.5. Annotation labels on the top (A) indicate samples from B/c naive mice (white) or B6 naive mice (gray). Rows were z-score transformed, and Euclidean distance was used to cluster the rows and columns in a hierarchical way. (B-C) Differentially expressed probes between B/c and B6 naive animals were identified to investigate transcriptional differences in genes related to phagocytosis (B) or NETs (C) at a steady state. Positive values in the x-axis indicate probes with a higher fold-change in B/c (right-pointing arrow), whilst negative values denote an increase in B6 (left-pointing arrow). The adjusted p-value (adj. p-value) was calculated using the Benjamini-Hochberg method (FDR) and a threshold of adj. p-value < 0.05 and log2 fold change > 1.5 was set to differentiate significant differential probes (red dots) from non-significant (gray dots). The list of phagocytosis and NET-related genes is described in the methods. n.s. Non-significant; (D-J) Expression values of differentially expressed probes found in the skin of B/c and B6 at steady-state conditions and 4 weeks p.i. The adjusted p-value (adj. p-value) was set to differentiate significant differential probes from non-significant. *p<0.05.

To further explore the balance between phagocytic activity and NET formation, we analyzed a publicly available microarray dataset of B/c and B6 mouse ears from naive mice (Accession number GSE56029) to evaluate the baseline expression of genes related to those pathways. A total of 1,489 genes were differentially expressed (adjusted p-value < 0.05 and log2 fold change > 1.5) in which 795 are increased in the B/c naive mice and 694 in the B6 naive mice. A list of genes related to phagocytosis obtained from the Gene Ontology database (GO:0006909, 356 genes) along with a manually curated list of genes related to NET release (51 genes) and phagocytosis (65 genes) obtained from established literature findings (S1 Table) were used to select probes from the differential analysis (Fig 5A). Seven probes targeting 6 transcripts related to phagocytosis were increased in B/c. In comparison, 8 probes representing 8 genes were increased in B6 skin (Fig 5B). Of note, one of the probes related to the *arhgap12* gene, which is involved in the formation of the phagocytic cup [28,32], showed one of the greatest fold change values whilst another probe for the same gene did not have a p-value above the threshold. Considering the NET-related genes, 4 probes representing 3 genes were significantly increased in B/c mice in contrast to no probe increase in the B6 naive mice (Fig 5C). Among them, we detected two probes for myeloperoxidase (*mpo*) gene significantly increased. These results indicate an important difference in myeloperoxidase expression between B/c and B6 naive mice, thus supporting evidence for a transcriptional program that favors NETs formation in B/c mice, which might impact neutrophil effector response to *L*. *major* invasion. Moreover, the notable difference in the expression of *arhgap12*, which is increased in B6 naive mice, could support the higher incidence of phagocytosis in the pool of neutrophils from B6 mice.

Furthermore, to explore potential upregulated pathways in the B/c and B6 skin microenvironment, we performed a Gene set enrichment analysis (GSEA) for enriched Gene Ontologies. Multiple probes mapping to the same transcripts were aggregated using the mean value, thus reducing the initial 1,489 differentially expressed probes to 1,271 genes (Fig 5C). A ranked file composed of gene names and log2 fold change values was given as input to cluster Profiler filtering results for pathways with FDR < 0.05. Thirty-five pathways were significantly enriched of which 7 increased in the B/c naive mice and 28 in the B6 naive mice (S9A Fig). As the B/c and B6 conditions were compared to each other, an increase in the enrichment score for B/c naive mice means, necessarily, a decrease in the same pathway for B6 naive mice and vice-versa (S9A Fig). Next, we conducted another gene set enrichment analysis, but this time evidencing the number of genes matching the ontology and the ratio of those genes considering all the genes in the ontology. Activated ontologies indicate enrichment in the B/c naïve, whilst suppressed represent ontologies enriched in the B6 naïve (S9B Fig). Of note, pathways enriched on B6 mice indicate a more robust immunity, including enrichment of genes involved in neutrophil and leukocyte migration, TNF production, and antigen presentation, whereas B/c showed a metabolic shift towards ATP and NADH metabolic processes. Finally, targeted transcriptome analysis done in the skin lesions 4wks p.i. with *L*. *major* of B/c and B6 (Accession number GSE56029) reveals that while B/c mice express significantly more genes related to NET release (*mpo*, *pi3ckg*) and pyroptosis (*il1b*, *nlrp3*), while B6 skewed cell death pathways towards apoptosis (*fasL*, *cas9*) and necroptosis (*mlkl*). We evaluated the frequency and relative numbers of intralesional neutrophils 4wks p.i. and we did not detect significant differences, which demonstrate that differential expression observed is this timepoint is not related to a bias created by a distinct neutrophil count (S10A and S10B Fig). Therefore, we highlight here important differences in the transcriptional program of these mice at a steady-state and during infection that could contribute to modulations in the neutrophil effector response signature of these mice.

**Fig 6:**
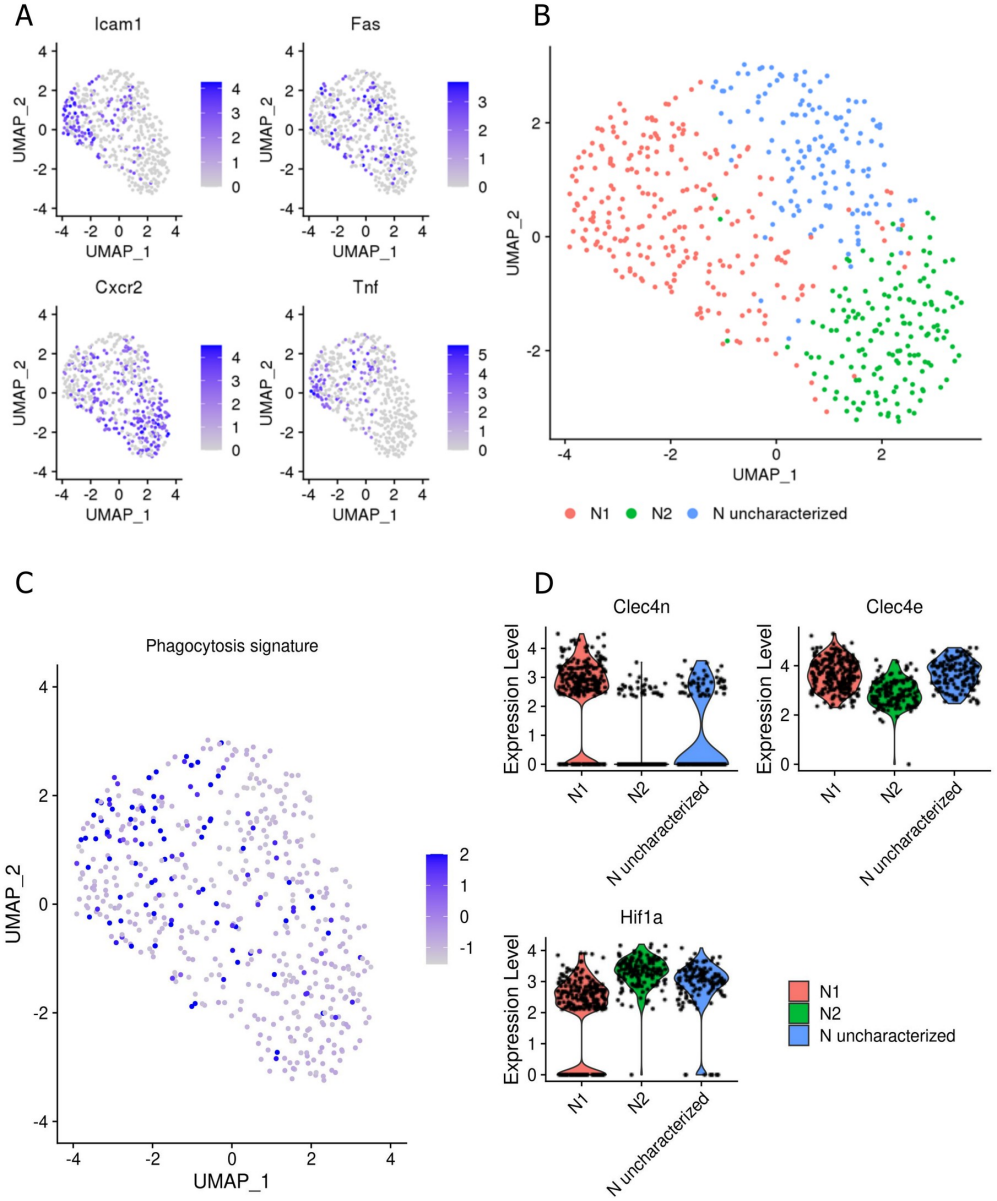
Identification of neutrophil subtypes related to phagocytosis and NET release. (A) scRNASeq data (GSE185253) was used to subset neutrophil cells according to their annotation verified by the expression of S100a9 and S100a8. We identified neutrophil subtypes by using four previously established markers of N1 (Icam1, Tnf, Fas) and N2 (Cxcr2) polarization based on DOI: 10.3389/fimmu.2020.00532. Each dot represents a cell and the color scales the expression level of the respective gene. (B) Spatial representation by Uniform Manifold Approximation and Projection (UMAP) of scRNASeq of neutrophil subclusters. Seurat workflow was used to find neighbors, clustering, and annotation. Three clusters were identified based on the marker genes from (A) and used to annotate the cells. (C) Meta signature consisting of 39 genes related to phagocytosis and expressed on neutrophils cells. Cells from N1 cluster presented higher scores of the meta signature than those of N2 cluster. (D) Analysis of differentially expressed genes between N1 and N2 clusters showed Clec4n and Clec4e as more expressed in N1 (adjusted p < 0.01), and Hif1a borderline higher in N2 (adjusted p = 0.11). The expression level is in reading counts and each dot represents a cell. RNA expression was imputed using Adaptively-thresholded Low Rank Approximation (ALRA) that deals with sparse matrices.

Interested in investigating whether transcriptional plasticity within neutrophil subpopulations has consequences for alterations in the microbicidal response, we accessed public scRNASeq data of B6 naive and *L*. *major* infected mice (GSE185253). From a total of 13,524 preprocessed cells, we analyzed 539 cells annotated as neutrophils. Based on clustering of these cells and on the expression of genes related to neutrophil polarization (*icam1*, *tnf*, *fas*, *cxcr2*) as previously described elsewhere [33] (Fig 6A), it was possible to annotate the subsets N1 (243 cells), N2 (158 cells), and a previously uncharacterized subset (138 cells) (Fig 6B). The three neutrophil subtypes identified revealed a distinct molecular signature, as demonstrated by the top 45 genes differentially expressed in each cluster (S11A Fig). Finally, we interrogated the expression of genes related to phagocytosis and NET release in those subpopulations. A meta signature of phagocytosis consisting of 39 genes revealed marked associations with the N1 cluster (Fig 6C). In contrast, the expression of the 24 genes related to NET release that we picked was low, with insufficient cells expressing the signature for further analysis. Nevertheless, two genes related to the phagocytosis signature, *clec4n* and *clec4e* were found upregulated in N1 (adjusted p-value < 0.01), whilst *hif1α*, involved in NET release [34], reached borderline significance (p-value < 0.05, adjusted p value = 0.11) in N2 (Fig 6D). The differential expression was verified by four statistical approaches built in the Seurat package to better assess the reliability of the findings and to increase the detection of potential genes related to phagocytosis and NET release. Most of the genes associated with phagocytosis or NET release signatures were below the default threshold of the package used for differential expression analysis, a recurrent limitation of scRNA-seq. The results of the findings, excluding non-evaluated genes, are depicted in S11B and S11C Fig. Lastly, the findings relate the expression of a meta signature of phagocytosis with the N1 profile and identify *clec4n* and *clec4e* as the main markers of this signature (Fig 6D). Therefore, indicating that within the heterogeneous pool of neutrophils, each subset possesses a unique transcriptional program that could contribute to shaping different phenotypic behavior in these cells.

### Neutrophil extracellular traps control parasite lymphatic drainage

**Fig 7:**
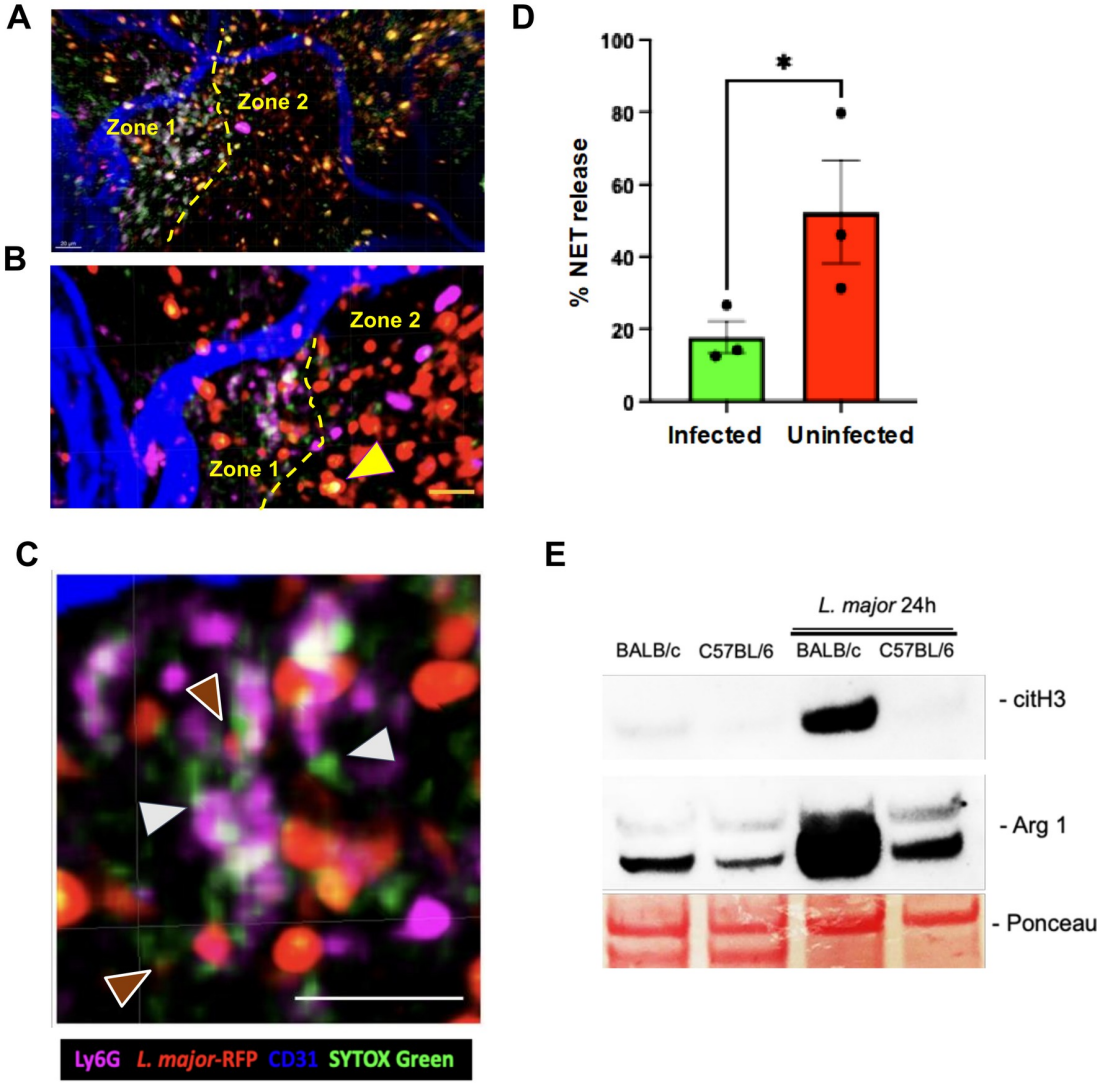
*In vivo* image shows zones of NET release spatially excluded from regions of phagocytosis. Mice were inoculated with RPF-expressing *L*. *major* to achieve a small focal infection in the ear pinnae. AF647-conjugated anti-Ly6G Fab antibody was injected i.v. 1 h later. Two hours after parasite inoculation, mice were anesthetized, injected with Sytox green i.v. and immediately imaged on a confocal microscope. Snapshots of time-lapse movies were taken 2–3 h p.i. (A, B) Representative images from infection sites in the skin of B/c mice. Pockets of vital NET release identified in zone 1 are spatially isolated from zone 2, where you find mostly infected cells. The yellow arrow points to a necrotic cell. (C) Representative image from infection sites shows pockets of NET formation within small clusters of cells, where white arrows point to NET-DNA fibers and brown arrows point for parasites trapped in the NETs. Bar = 20μm. (D) Quantification of the percentage of NET release among infected and non-infected cells including zones 1 and 2. (E) Immunoblot for citH3 histones and Arg1 from whole skin tissue lysate obtained from non-infected and infected ears of B/c or B6 24 h p.i. Results are mean±SEM or representative data of 2–3 independent experiments.

To visualize and validate NET formation *in vivo*, we conducted intravital confocal imaging in the ear pinnae to monitor neutrophil infiltration and extracellular trap formation during intradermal *Leishmania* infection in B/c mice. We injected 10^5^ RFP-expressing promastigotes of *L*. *major* in 10 μL in order to get a focal point of infection. To monitor Ly6G^+^ cells, we generated and purified a Ly6G Fab fragment, which was conjugated to Alexa Fluor 647, in order to prevent antibody-mediated neutrophil cell death. We confirmed the purity of our preparation (S12A Fig), binding (S12B Fig) and specificity (S12C Fig) of our Ly6G Fab fragments. Additionally, Sytox Green staining was used to detect NET formation in the tissue. Time-lapse imaging of the infection site shows pockets of early NET release with Ly6G^+^ cells surrounded by diffuse or thin filaments of Sytox Green staining (Fig 7A). Zone 1 and 2 distinguish regions of predominant NET release from sites with mostly infected cells and dead cells with condensed chromatin (yellow arrow head), respectively (Fig 7B). NET connected to neutrophil (white arrows) and the *Leishmania* caught in the NET (brown arrow) are observed (Fig 7C). We also quantified the percentage of NETing neutrophils among infected and non-infected cells, and we observed an inverse correlation of NET release with internalization of parasites (Fig 7D). Finally, we compared citH3 in skin tissue lysates of both mice strains by immunoblotting at 24 h post-infection, and we observed significant upregulation of this NET biomarker in B/c, whereas citH3 was absent in B6 skin lysate (Fig 7E). Arg1 was used as a control to validate immunological response skewed towards Th2 immunity in BALB/c mice. Moreover, similar differences were observed upon infection with *L*. *amazonensis* using the whole IgG Ly6G antibodies (S13 Fig and S2 and S3 Videos).

**Fig 8:**
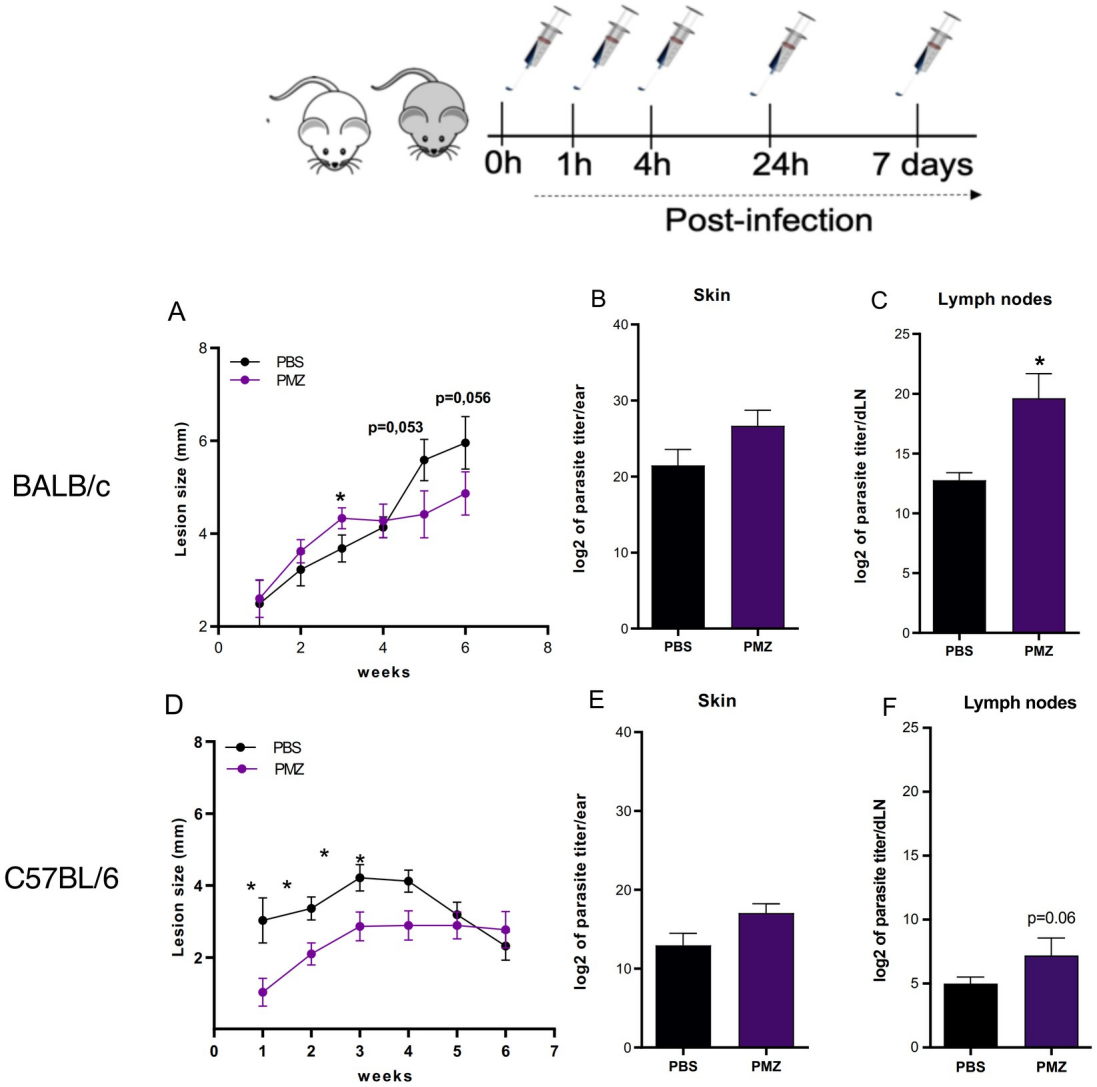
NETs regulate parasite drainage to lymph nodes. The schematic figure shows the protocol of DNAse treatment adopted. (A, D) Progression of lesion size over time among B/c and B6 injected intradermally with 10^6^ metacyclics of *L*. *major* in the ear pinnae. (B, E) Parasite load in the skin lesion 6 weeks post-infection and (C, F) parasite load in the draining lymph nodes from B/c and B6 mice, respectively. Pulmozyme (PMZ) treated animals (purple columns or line). Results are mean±SEM of n = 4–6. Results are compilation of 2–3 independent experiments. *p<0.05.

To investigate the biological impact of NET formation *in vivo*, B6 and B/c mice were injected intradermally in the ears with 10^6^ metacyclic promastigotes of *L*. *major* in combination or not with alpha dornase (Pulmozyme), a biosynthetic form of DNAse I (Fig 8). Mice were treated repeatedly with PBS or alpha dornase i.v. to assure digestion of NETs generated by the continuous neutrophil influx [20,35,36]. Our results demonstrated that treatment with alpha dornase in B/c mice promoted a transient increase in lesion diameters reaching statistical significance at 3 weeks post-injection, but no differences were observed at later time points compared to PBS (Fig 8A). Conversely, alpha dornase administration increased disease tolerance in B6 mice compared to vehicle alone (Fig 8D). No differences were observed in parasite loads found in the skin among groups of both strains (Fig 8B and 8E). Remarkably, NETs digestion resulted in the enhancement of parasite lymphatic drainage in both strains, while alpha dornase treatment increased 5-fold the number of parasites in the lymph nodes of B6 mice, the same treatment resulted in a 1351-fold increase in B/c mice compared to control (Fig 8C and 8F). Additionally, we have induced higher or lower nucleotidase activity in *L*. *amazonensis* by culturing them in low-phosphate (LP) or high-phosphate (HP) conditions, respectively (S14A Fig). Subcutaneous injections of HP or LP parasites in the footpads of B/c mice resulted in increased retention of parasites in the footpad of animals infected with HP parasites (S14B Fig), whereas higher nucleotidase activity enhanced lymphatic drainage of parasites, which provides orthogonal validation that NET digestion facilitates lymphatic drainage of parasites (S14C Fig). Altogether, our data support that the formation of NETs predominates at the site of infection in susceptible mice, and suggest that NET formation might directly modulate the early antigen load in draining lymph nodes, which could have implications in shaping host adaptive immunity.

## Discussion

Massive neutrophil influx starts at the inoculation site immediately after sand fly bites, and egested metacyclics that are promptly phagocytized by both neutrophils and resident macrophages [35,37]. Depletion of neutrophils results in different outcomes in experimental models of *L*. *major* infection, which is conditioned to the experimental protocol used and host genetic background [36,38]. Phenotypic distinctions in these granulocytes might be extrinsic, related to the microbiome and to the immunological microenvironment created during each host response to infection, or intrinsic, such as genetic factors predisposing those neutrophils to respond differently, or even a combination of both. Previous evidence has demonstrated that *L*. *major* induces opposed responses on neutrophils derived from susceptible and resistant mice regarding cytokine production such as IL-12p40, IL-10, and TLRs expression [5], while levels of elastase degranulation have also been shown to differ in quiescent neutrophils derived from these mice [13]. Accordingly, we have deepened those observations by showing that BMN from susceptible mice is more likely to form NETs at basal state and respond to *L*. *major* than neutrophils from resistant animals. Though basal NET formation differences were neutralized by inflammatory priming, responsiveness to parasites was maintained in inflammatory neutrophils and reproduced on mice from a different animal facility and laboratory setting, confirming the robustness and reliability of this phenotype across different laboratories and discarding any role of microbiota on this phenotype. Furthermore, the injection of promastigotes in the peritoneal cavity also resulted in the release of significantly more NET-DNA in the susceptible background than resistant. However, neutrophils were not the major population in the inflammatory influx recruited to the peritoneal cavity by the parasites, which agrees with previous reports [36]. Thus, DNA extracellular traps production might be globally enhanced on immune cells in B/c background compared to B6.

Enhanced neutrophil elastase activity was detected in cultures of neutrophils obtained from B6 mice, at basal levels, compared to B/c [13,39]. We observed a similar trend under our experimental conditions during the first 2 h of incubation, and to a lesser extent after 4 h. However, in the presence of promastigotes, while B/c neutrophils increased the release of elastase in a parasite-dependent ratio, levels of elastase activity in B6 neutrophil cultures remained unchanged regardless of the parasite ratio. Fluorescence microscopy revealed that B6 mice have significantly diminished elastase overlaying NET fibers, while B/c mice formed NET fibers enriched in this serine-protease. Although we observed more elastase attached to NET fibers on susceptible mice, we cannot exclude the possibility that some of the elastase found associated with these fibers might be a result of unsystematic extracellular adsorption of granular proteins to NET fibers on our cultures, whether this process is translatable to *in vivo* setting remains to be addressed. Yet, evidence generated by proteomic analysis has demonstrated that NETs composition vary in response to different stimuli [40,41]. Although NETs were not measured, it has been shown that sequential intranasal application of LPS and fMLP, resulted in significantly higher MPO and elastase activity recovered from bronchoalveolar lavage fluids of B/c compared to B6 mice [42]. This suggests that both enzymes release by neutrophils is a rather tightly regulated process conditioned by genetic background and the type of stimulation, which could ultimately impact the repertoire of granular proteins decorating NET fibers.

We could not detect any ROS generation induced by *L*. *major* on neutrophils in both mice. Data regarding ROS production by neutrophils in response to *Leishmania sp*. infection is still controversial. While it has been reported that *L*. *major* inhibits ROS production by PMA-activated neutrophils, some other studies showed that mouse neutrophils enhance respiratory burst in response to *L*. *major in vitro* but fail to induce ROS in human neutrophils [43], whereas ROS production in human neutrophils has been reported with other *Leishmania* species [44,45]. Although classical NET formation by certain stimuli requires ROS generation [46], we describe here that NADPH oxidase is not required for *L*. *major* induction of NETs in mice and, in fact, it has been demonstrated that NET formation can occur independently of ROS generation or even ensue under ROS suppressive conditions [47]. Nevertheless, important differences exist between murine and human neutrophils, hence a potential role of *L*. *major* suppressing ROS production in murine neutrophils require further investigation. Finally, differences in probe specificity and sensitivity [48] may also account for the results we obtained, and recent evidence have demonstrated the requirement of opsonization for ROS induction in mouse neutrophils [49].

We describe here that NE expression and its mobilization to the nucleus are central to induce NET formation as elastase knockout mice did not respond to promastigotes compared to wild-type neutrophils. Accordingly, human neutrophils also require neutrophil elastase activity in order to produce NETs in response to *L*. *amazonensis* [45].

*In vivo* imaging of neutrophils at the infection site showed a rapid swarming of cells and a large proportion of parasite-infected granulocytes in the infiltrate in a B6 background [37]. However, the profile of neutrophil effector response in B/c mice at the infection site had been poorly investigated. We report higher parasite uptake by B6 neutrophils compared to B/c mice *in vitro* and *in vivo*. Hence, the inability of neutrophils to internalize parasites right away on B/c mice might shift infection to other permissive cell types that support parasite replication including inflammatory monocytes, macrophages and fibroblasts [4,35,50], which might further contribute to increased susceptibility to infection and latency in these mice. Here, we have shown that neutrophils from elastase knockout mice possess higher phagocytic capacity compared to wild-type. Indeed, phagocytosis activation has been shown to antagonize nuclear translocation of NE, and data supports that neutrophils that had phagocytosed apoptotic bodies were unable to release NET [26,27]. Previous observations also indicated that the phagocytosis of promastigotes or NET release is a dichotomous response within the pool of human neutrophils isolated from the peripheral blood [51]. Hence, we have shown here that elastase is a central regulator of neutrophil effector response. The mechanisms by which elastase regulates neutrophil phagocytosis remains to be elucidated. The ability of NE and MPO to promote chromatin decondensation [52] may contribute to epigenetic modifications on neutrophils that could shape the program of these cells as well. Indeed, we observed differences in the levels of intranuclear MPO in neutrophils from B/c and B6 at steady state.

Elastase has been described as an immune modulator agent that activates a leishmanicidal state on macrophages via TLR4 at nanomolar doses [13,31]. However, high doses of elastase have been shown to promote tissue injury [53–55]. Previous reports have also demonstrated that elastase is toxic to bacteria and worms [56–57]. We have shown here that elastase could directly kill *L*. *major* promastigotes, through a yet uncharacterized mechanism that affects parasite motility. Presence of human elastase inside parasites suggests that this enzyme may have the potential to actively disrupt parasite homeostasis by targeting proteins in the cytoplasm. It has already shown that elastase digest cytoskeleton filaments [58]. Furthermore, we propose that low concentrations of elastase may allow intercellular host communication and are tolerable by parasites, which allow them to resist the toxicity of NETs derived from B6 mice, whereas high concentrations of the enzyme mounted by B/c extracellular response are potentially toxic to the parasites. Yet, most of the promastigotes evaded elastase-mediated NET killing by expression of ISP2, which demonstrates the inadequacy of the extracellular response mounted by B/c neutrophil to control infection, and is in line with the susceptible phenotype of this mice.

The “Trojan Horse” model proposes that infected neutrophils undergoing apoptosis shield promastigotes and serve as carriers of parasites to mononuclear cells, mainly tissue resident macrophages, the main cell of parasite replication [22,35]. Previous observations have confirmed that infected neutrophils sorted from ear dermis carried viable parasites that were able to recover [37], while intravital imaging have shown evidence of efferocytosis of infected neutrophils [35]. Despite higher neutrophil phagocytosis in the B6 background, we recovered viable intracellular promastigotes in neutrophils from both mice, suggesting ineffective intracellular parasite killing. However, growing evidence in the literature points out that neutrophils are a heterogeneous population with distinct pools of cells that differ in phenotype and function [38]. Hence, intrapopulation variability in the continuous influx of neutrophils may still affect the fate of internalized parasites, allowing alternative outcomes that require further studies. Microarray datasets of B/c and B6 naive mice evidenced significant differences among the expression profile of those strains. On one hand, it evidenced the upregulation of myeloperoxidase in B/c naive mice in comparison to B6 [42], which has an important role in NET formation as nuclear translocation of MPO is critical to promote chromatin decondensation prior to NET release [52]. On the other hand, Arhgap12, a member of the RhoGAP family required for the process of phagocytic cup formation, showed differential expression in B6 mice [28,32]. Whether metabolic shifts in neutrophils from these mice during infection can also affect neutrophil response to promastigotes remains to be elucidated. Metabolic reprogramming has been demonstrated in neutrophils during NET formation in response to amyloid fibers to fuel NADPH oxidase activity by shifting the cell metabolism towards the pentose phosphate pathway [59]. Mitochondrial metabolic dysfunction also affects phagocytic capacity of dendritic cells, corroborating that metabolic alterations may affect neutrophil microbicidal response [60]. We observe a global enrichment of genes related to antigen processing and presentation on resistant mice skin transcriptome at steady-state, whereas B/c skin transcriptome showed a metabolic shift towards ATP and NADH metabolic processes. Those intrinsic changes may contribute to shape neutrophil effector response in *L*. *major*-infected lesions, which displayed upregulation of genes involved in necrosis and apoptosis in the lesions of B6, whereas lesions of B/c skewed neutrophil response towards NET extrusion and pyroptosis. In fact, prolonged inflammasome activation has been associated with IL-1β mediated pathology in cutaneous leishmaniasis, suggesting that neutrophil microbicidal phenotype may also contribute to drive tissue damage on susceptible background [61]. Of note, we cannot exclude the influence of other extrinsic signals coming from outside of the immune system compartment that add another layer of complexity in plasticity of neutrophil phenotypes. Moreover, despite enrichment of these genes in granulocytes, we cannot rule out the quantitative contribution of skin resident neutrophils versus other resident myeloid cells in the alterations we saw on skin transcriptome in naïve skin.

The continuous influx of neutrophils reaching the inflammatory site encounters a distinct set of immunomodulatory signals as the inflammatory reaction evolves, and along the distinct zones within the tissue as well, which altogether may contribute in the polarization of distinct neutrophil subpopulations [38]. We have observed, by scRNA-seq, three distinct subpopulations of neutrophils during the chronic phase of *L*. *major* infection in B6 mice, which corroborates the existence of transcriptional plasticity among neutrophils. Furthermore, we identified a subpopulation, N1, with a phagocytosis transcriptional signature on B6 mice, which reinforces that a specific transcriptional signature determines the type of microbicidal response in these cells. Our inability to identify a specific microbicidal signature on the other subpopulations might be due to the prevalence of N1 subpopulation on B6 mice and, secondly, to the fact that we did not had access to B/c scRNA-seq, on which we should expect a shift in the distribution of these subpopulation. Despite this, the findings related to differential expression of *Hif1a* in N2 require further investigation, as *Hif1a* expression is one of the few genes whose activation has been directly associated with NET formation induced by LPS [34].

We obtained evidence of NET formation *in vivo* by intravital imaging at the infection site of B/c mice following intradermal injection of metacyclic promastigotes, and differences in the levels of histone citrullination in total skin tissue lysates from B/c and B6 validates dichotomy in NET formation. Previous reports have shown that Lundep, a sandfly salivary gland endonuclease, may destroy NETs when co-injected with *L*. *major* [21]. Although this mechanism may impact NET production at the site of the bite, it is improbable that Lundep would remain active in the tissue after saliva dissipation and the continuous influx of neutrophil during acute and chronic stages of inflammation. Indeed, NETs have been detected in lesions of patients with cutaneous leishmaniasis [18,62]. Hence, NET formation during the prolonged neutrophil infiltration would likely bypass the transient effect of Lundep.

Spatial separation of NET producing neutrophils from infected cells suggests that cues present in the tissue microenvironment that impregnate the extracellular matrix may create a microenvironment that shapes polarization of neutrophils towards an intracellular (phagocytosis), or an extracellular response (NET formation). Whether a fixed set of neutrophil subpopulations circulating in the blood selectively respond to various chemotactic signals leading to regional separation of subsets in the tissue remains to be addressed. The tumor microenvironment is a well-studied milieu where neutrophils encounter signals that drive N2 polarization [38]. Recent evidence has shown that human neutrophils can be polarized *in vitro*, and that N2 phenotype favors *L*. *donovani* replication [33]. Our results suggest that N2 polarization could be related to NET formation, since this subset displayed increased levels of *Hif1a*. The relative contribution of signals coming from promastigotes, sand-fly saliva, microbiota or the host itself to drive neutrophil polarization in the context of *Leishmania* infection remains to be elucidated.

Digestion of NETs through DNAse treatment resulted in a robust increase in the lymphatic drainage of parasites to the ear lymph nodes of both mouse strains without affecting parasite loads in the skin. Previous reports have shown the importance of NETs to contain *Pseudomonas aeruginosa* brain invasion and *S*. *aureus* systemic dissemination [63,64]. The profile of cytokines produced by dendritic cells is regulated by the density of antigens in the microenvironment during antigen presentation, which might be affected by the density of parasites that reach the draining lymph nodes [65,66]. On the other hand, presence of parasites in the lymph nodes may also have a suppressive effect on T cells since the high parasite load of *L*. *donovani* in the bone marrow of VL patients correlated with an increase in the frequency of IL-10 and FOXP3^+^ regulatory T cells [67]. Therefore, regulation of the parasites lymphatic drainage by NETs could affect T cell differentiation and response. Hence, though increased tolerance to disease in the B6 background upon DNAse I treatment requires further investigation, this phenomenon could be potentially related to alterations in the adaptive response of these mice.

In conclusion, transcriptional variations found in neutrophils of resistant and susceptible mice mirrors functional alterations in the effector response of neutrophil subpopulations. NETs contribute to contain parasite drainage beyond the primary site of infection, which highlights the importance of this response of the host in the combat of infection through other mechanisms than just direct parasite killing. Finally, plasticity on neutrophil effector response indicates these cells may also possess characteristic response signatures that may have potential implications for the establishment of infection, and affect subsequent immunological events in the tissue. This could contribute to explain the immunological dichotomy in the immune responses of these mice to *L*. *major* infection.

## Materials and methods

### Ethics statement

Procedures and animal protocols were approved by the Committee for Animal Use of the Universidade Federal do Rio de Janeiro (CEUA-UFRJ) permit numbers 127/15 and IMPPG022. Alternatively, some of the mice used in this work were used under a study protocol approved by the National Institute of Allergy and Infectious Diseases—Animal Care and Use Committee (protocol number LPD 68E). All aspects of their use were monitored for compliance with The Animal Welfare Act, the PHS Policy, the U.S. Government Principles for the Utilization and Care of Vertebrate Animals Used in Testing, Research, and Training, and the NIH Guide for the Care and Use of Laboratory Animals.

### Mice

BALB/c (B/c) and C57BL/6 (B6) female mice (6–10 weeks) were obtained from the Núcleo de Animais de Laboratório (Universidade Federal Fluminense, Rio de Janeiro, Brazil) or Taconic Biosciences (Maryland, USA). All performed procedures were in strict accordance with the Brazilian animal protection law (Lei Arouca number 11.794/08) of the National Council for the Control of Animal Experimentation (CONCEA, Brazil). *Tlr4*^*-/-*^ mouse lineage (in the C57BL/6 genetic background) was obtained from Dr. S. Akira (Osaka University, Japan). *Tlr4*^*-/-*^ and C57BL/6 mice were maintained at the Laboratório de Animais Transgênicos (LAT IBCCF, UFRJ, RJ, Brazil). All mice were kept under controlled temperature and light conditions and allocated in Ventilife mini-isolators (Alesco, Brazil). gp91phox^-/-^ mice were kindly provided by Dr. Leda Quercia Vieira (Universidade Federal de Mi9nas Gerais, MG, Brazil) and neutrophil elastase knockout mice by Dr. Ana Paula Lima.

### Parasites

*Leishmania major* (MHOM/IL/80/Friedlin) and *Leishmania amazonensis* (WHOM/BR/75/Josefa) promastigotes were maintained at 26°C in Schneider’s Insect medium (Sigma) supplemented with 10% heat-inactivated fetal calf serum (FCS), 2% human urine and 40 μg/mL gentamicin. Stationary-phase promastigotes were obtained from 5- to 6-day-old cultures and washed two times with PBS and kept on ice till use. Metacyclic forms were purified using Ficoll gradient [68]. Alternatively, *Leishmania amazonensis* was cultured under conditions of high (HP) or low (LP) concentrations of phosphate (Pi) as described previously to modulate nuclease activity [20]. Alternatively, *Leishmania major* ISP2/3 were grown in modified Eagle’s medium (designated HOMEM medium) supplemented with 10% heat-inactivated fetal calf serum (FCS) at 25°C and transfectants were generated as previously described [31].

### Parasite labeling

Promastigotes were labeled with CellTrace CFSE Cell Proliferation kit (Invitrogen) according to manufacturer’s instructions. Briefly, stationary-phase parasites were washed twice with PBS and incubated with 1uM of CFSE in serum-free media at 35°C, 5%CO_2_. After 30 min, cells were kept on ice with 20% of FBS to remove unbound CFSE for 15 min. Cells were washed twice with cold serum-free RPMI and kept on ice till use. Alternatively, purified metacyclic promastigotes (10^7^cells/mL) were stained with 2 μM of PKH26 in PBS at room temperature with a gentle shake every minute. After 5 min, cells were kept on ice with 20% of FBS to remove unbound dye for another 5 min. Cells were washed twice with cold PBS and kept on ice protected from light before use.

### Neutrophil isolation

Neutrophils were obtained from murine bone marrow as previously described [69]. Briefly, total cells obtained from flushing femurs and tibias were separated on a discontinuous Percoll gradient (58%, 65%, and 72% v.v.; GE Healthcare, Little Chalfont, UK), and neutrophils were enriched between 65% and 72% solutions (74.5% ± 2.59% Ly6G^+^). Alternatively, peritoneal cavity neutrophils were collected 4 h after i.p. inoculation of 1.5 mL of 90 mg/mL casein enzymatic hydrolysate (Sigma), and >85% Ly6G^+^ neutrophils were obtained. Additionally, 2x10^6^ metacyclic promastigotes were inoculated in the peritoneal cavity and after 3 h, the inflammatory infiltrate (percentage of Ly6G+ cells: 36.8±8.7; 42±7.8 for B/c and B6, respectively) was collected as above. Additionally, highly purified neutrophils were obtained from bone marrow total cells by negative selection with Neutrophil Isolation kit (Myltenyi Biotec).

### Tissue processing

10^6^ purified metacyclics of *L*. *major* labeled with PKH26 were injected intradermally in a final volume of 10 μL of PBS. After 3 h, mouse ears sheets were separated and cut in small pieces and digested with 3 mg/mL collagenase type IV (Sigma-Aldrich) for 90min at 37°C and 5% CO_2_. Digested tissue pieces were further homogenized through a 70um cell strainer to obtain a single cell suspension and washed with 1mL of ice-cold PBS.

### NET quantification assay

Neutrophils (5 x 10^5^ cells/ well) were either left untreated (Nil) or incubated with increasing ratios of *Leishmania sp*. promastigotes (10 neutrophils: 1 parasite, 1:1, 1:5) for 2 or 4 h at 35°C and 5% CO_2_. Culture supernatants were collected and extracellular DNA and elastase activity were measured using Quant-IT dsDNA Picogreen kit (Invitrogen, California, USA) and the fluorogenic substrate N-Methoxysuccinyl-Ala-Ala-Pro-Val-7-amido-4-methylcoumarin (Sigma-Aldrich), respectively. Additionally, the kinetics of NET formation was measured every 10 min with 5 μM Sytox Green (Invitrogen, California, USA). Fluorescence was detected on a SpectraMax Paradigm microplate reader (Molecular Devices, California, USA). Data are presented as arbitrary units (AU) or fold increase over control. Elastase/DNA ratio was calculated as Elastase activity (AU)/DNA (AU).

### Fluorescence microscopy

Neutrophils (1 x 10^5^ cells/well) were adhered onto poly-L-lysine coated coverslips and incubated with *Leishmania sp*. promastigotes (1:5) at 35°C and 5% CO_2_. After 4 h, cells were fixed with 4% formaldehyde and slides were stained with DAPI (10 μg/mL, Sigma-Aldrich), Hoescht 33342 (1:10.000), anti-neutrophil elastase rabbit pAb (1:250) (Calbiochem, California, USA) followed by anti-rabbit-Alexa488 (1:500; Invitrogen, California, USA) and anti-CD63-APC (1:150; Biolegend), anti-MPO (1:100; 8F4, Hycult). Images were captured using a Zeiss Axioplan-2 microscope (Oberkochen, Germany) equipped with a Color View XS digital video camera. Alternatively, for elastase binding assays to parasites, *L*. *major* WT, Δ*isp2/3*, and Δ*isp2/3*: *ISP2/3* promastigotes were incubated with 10 μg ml^-1^ recombinant human neutrophil elastase or in medium alone for 2 h at 35°C and 5% CO_2_. Parasites were then fixed in 1% formaldehyde, left to adhere to poly-L-lysine coated chamber slides (Lab-Tek), and further permeabilized with 0.1% Triton X-100 followed by addition of 0.1 M glycine then blocked in 0.1% (v/v) Triton X-100, 0.1% (w/v) BSA, in PBS for 18 h at 4°C. Slides were stained with anti-neutrophil elastase rabbit pAb (1:200) followed by goat anti-rabbit Alexa Fluor 488-conjugated antibody (Invitrogen) (1:2000). Slides were mounted with ProLong Gold antifade (Invitrogen). Images were obtained on Zeiss Cell Observer SD automated microscope integrated with Yokogawa confocal spinning disk. Imaging processing and analysis were done in Image J or Imaris software. To quantify subcellular distribution of MPO, cells were counted on FiJi and to create surfaces for nucleus (DAPI signal) and MPO signal (stained in red) images were processed with Surface colocalization extension on Imaris. MPO intensities in nucleus, cytosol, and colocalization of MPO with DNA were measured on Imaris. 3D reconstruction from compiled z-stack images was done on Imaris.

### Flow cytometry and cell sorting

Neutrophils (5 x 10^5^ cells/ well) were either left untreated (Nil or -) or incubated with increasing numbers of *Leishmania sp*. promastigotes (10 neutrophils per 1 parasite, 1:1, 1:5) for 2 or 4 h at 35°C and 5% CO_2_. Neutrophils were identified by anti-Ly6G-APC or anti-Ly6G-FITC (Biolegends) and further analyzed by staining with anti-CD11b-PE Ab (Biolegends), anti-CD63-APC (Biolegends) and analyzed on a MACSQuant flow cytometer (Miltenyi Biotec). For cell sorting, infected RFP^+^ and uninfected RFP^-^ inflammatory neutrophils (CD11b^+^SSC-A^high^) were purified using a BD FACSAria IIu Cell Sorter on cells recovered from peritoneal lavage fluid 3h post-injection with casein hydrolyate i.p. and 2h after injection with 2×10^6^ *L*. *major*-RFP stationary phase promastigotes i.p. Sorted populations were kept on ice and washed once in cold PBS and immediately ressuspended in modified Eagle’s medium (designated HOMEM medium) supplemented with 10% heat-inactivated fetal calf serum (FCS) and 0,5% hemin and kept at 25°C. After 48 hours, parasite loads were quantified by limiting dilution and numbers were normalized based on the number of events obtained from the cell sorter for each sample. All flow cytometry data were analyzed with FlowJo Software 10.0.8.

### ROS generation measurement

Neutrophils (5 x 10^5^ cells/ well) were labeled with 1 μM of the fluorogenic probe Dihydrorhodamine 123 (Sigma-Aldrich) and incubated or not with *Leishmania sp*. promastigotes (1:1 or 1:5) for 15 min. PMA (100 nM) was used as a positive control. Fluorescence intensity was monitored for 90 to 100 min every 5 min at 35°C using a SpectraMax Paradigm microplate reader.

### NETs killing assay

Casein-elicited inflammatory neutrophils (6-7x10^6^) were stimulated with formaldehyde-fixed promastigotes (1:5) for 4 h and NETs-enriched supernatants were generated. NETs-enriched supernatants (2–3 μg DNA), pre-treated or not with 100 U/mL DNAse (Promega) or 20 μg/mL of elastase inhibitor II (Calbiochem) for 30 min were incubated overnight with promastigotes (2 x 10^6^) at 35°C, 5% CO_2_. Motile promastigotes were counted in a Neubauer chamber to assess their viability.

### Elastase killing assay

*L*. *major* promastigotes were incubated with 2.5 or 5 μg/mL of purified native elastase from human neutrophils (Sigma-Aldrich) for 2 h at 35°C, 5% CO_2_. When indicated enzyme activity was blocked by 30 min pre-treatment with 10 or 20 μg/mL of elastase inhibitor II or antibody anti-elastase (1:100; 1:200). Parasite viability was assessed by flow cytometry using propidium iodide staining (1μg/ml; Sigma-Aldrich) or parasite viability was measured by XTT assay (Sigma-Aldrich) according to the manufacturer’s instructions.

### Quantification of 3’-Nucleotidase activity

Promastigotes (1 × 10^7^ cells/mL) were incubated for 1 h at 25°C in a mixture containing 116 mM NaCl, 5.4 mM KCl, 5.5 mM glucose, 50 mM HEPES-Tris buffer (pH 7.0), and 3 mM 3’-AMP as the substrate. The Pi content released was quantified by spectrophotometer at 650 nm using the Fiske-Subbarow reactive mixture [70]. The concentration of Pi was determined by using a Pi standard curve for comparison. The ecto-3’-nucleotidase activity was calculated by subtracting the nonspecific 3’-AMP hydrolysis measured in the absence of cells [20].

### Experimental models of cutaneous leishmaniasis

Mice were intradermally injected in the ear with 10^6^ *L*. *major* metacyclic promastigotes diluted in PBS or Pulmozyme (dornase α) in 15 μL final volume. After inoculation, PBS or Pulmozyme (20 mg/kg, Roche) were administered i.v. 1h, 4 h, 24 h, and 7 days according to neutrophil influx peaks [15,16]. Lesion size was weekly measured and parasite load quantified after six weeks in the ear and in draining lymph nodes by limiting dilution assay. Alternatively, B/c mice were injected subcutaneously in the footpad with 10^6^ HP or LP promastigotes of *L*. *amazonensis* (WHOM/BR/75/Josefa). After 26 days, parasite loads were quantified in the site of lesion and draining lymph nodes by limiting dilution.

### Intravital imaging

Intravital imaging was performed as described previously with minor modifications [37]. Mice were anesthetized with a subcutaneous injection of mixed ketamine and xylazine in sterile saline (60 mg/kg and 15 mg/kg, respectively). Ears were shaved to minimize image interference of hair follicles and anesthetized mice were then injected intradermally with a minimum volume (approx. 10 μL) of an RFP-expressing *L*. *amazonensis* suspension (10^7^ parasites/mL in PBS). One hour after parasite inoculation, mice were injected intravenously with 10 μL (2 μg) of BV421-conjugated anti-Ly6G antibody (BD Biosciences). One hour after antibody injection (2 h after parasite inoculation), mice were anesthetized as before and injected i.v. with 10 μL (5 nM) of Sytox-green nucleic acid stain (Thermo Fisher Scientific). Mice were then immobilized on a custom-made imaging platform, with the ventral side of the ears resting on a microscope coverslip. Mice were immediately imaged using a Nikon Eclipse Ti with an A1R confocal microscope. Images were acquired with the Nikon NIS Imaging software. Differences between experimental conditions were compared by calculating the ratio of the area occupied by DNA (excluding nuclei) associated with neutrophils (DNA ROI) to the area of the neutrophils (blue mask) in the image. To define the blue mask, BV421 channel was thresholded using the Otsu method from the threshold plugin drop-down menu and a selection was made. To define the DNA ROI, the green (image obtain from 488 channel) was thresholded within the range of 500 to 2500 AUs, where the upper threshold was selected to eliminate the signal from nuclei. A green mask was created from the threshold and a selection was made. Using the ROI manager macro tool, the selections from the blue and green masks were added as ROIs. Next, an ‘OR’ operation was performed on the blue and green masks to obtain an ROI where the two masks overlap to produce the DNA ROI. Finally, the area (in pixels) of the DNA ROI and the blue mask was quantified and a ratio was produced by dividing the area of the DNA ROI by the area of the blue mask to estimate the production of NETs per neutrophil.

### Western-blot

Mice were intradermally injected in the ear with 10^6^ *L*. *major* metacyclic promastigotes diluted in PBS in 15 μL final volume and compared to naive skin. Whole skin tissue lysates were obtained and processed for western-blot as previously described [16]. Briefly, mouse ears were processed in RIPA lysis buffer supplemented with Halt Protease Inhibitor Cocktail (ThermoFisher) combined with mechanical dissociation with Medimachine System (BD). We quantified protein concentration from the soluble fraction that was collected and used for western-blot analysis.

### Data accession and microarray analysis

Quantile-normalized array data was retrieved at the Gene Expression Omnibus database (accession number GSE56029) using GEOquery v2.58. The analysis followed the recommendations for BeadArray analysis and used the illuminaMousev2.db for probe annotation. Probes with no quality information or annotated as “Bad” were removed. The limma package v3.46 was used to identify differentially expressed probes between B/c naive and B6 naive conditions and to calculate the adjusted p-value using the Benjamini-Hochberg method (“BH” or “FDR”). A threshold of adjusted p-value < 0.05 and log2 fold change > 1.5 was used to set differentially expressed probes. A list of mice genes related to phagocytosis was obtained at the Gene Ontology database (GO:0006909) in addition to a manually curated list of NETs and phagocytosis-related genes (S1 Table). The Gene-set enrichment analysis (GSEA) was performed on Gene Ontology annotations using a ranked file for all probes containing gene name and log2 fold change as input for the cluster profile package v3.16.1. Multiple probes targeting the same gene were aggregated using the mean of the probes. Significantly enriched pathways were selected applying a threshold of FDR <0.05. Analysis was conducted in the R environment version 4.0.3. The cluster profile package v3.16.1 was used to perform gene-set enrichment analysis (GSEA) of gene ontologies considering B/c naive as reference. Probes mapping to the same gene were aggregated using the median of log2 fold change value. The parameters used in the GSEA analysis included a p-value cutoff of 0.001, minGSSize = 5, maxGSSize = 800, and BH method to adjust p-value. Ontologies matching the keywords immune, MHC, cytokine, chemokine, T cell, tumor necrosis factor, ATP metabolic, chromatin, leukocyte,Fc, glycolytic, lactate, NADH, autophagy, phagocytosis, inflammatory, chemotaxis, neutrophil, or innate, were kept.

### Analysis of public single-cell transcriptomics of C57BL/6NCr mice

scRNASeq data were retrieved at GEO with the identifier number GSE181720. The standard workflow from Seurat v4 was used to subset the cells annotated as neutrophils, normalize the counts, scale data based on all genes, extract the principal components and run Uniform Manifold Approximation and Projection (UMAP) for 2D projection of the cells. The neutrophil clusters were identified by running Seurat functions FindNeighbors with default parameters and FindClusters with a resolution of 0.4. The meta signature of phagocytosis-related genes was obtained from the original list of 68 filtering for a minimum count of 10 in all neutrophil cells to exclude lowly expressed genes. The search for differentially expressed genes between N1 and N2 clusters was conducted using FindMarkers with a minimum percentage of at least 25% of cells expressing the tested genes. RNA expression was imputed using Adaptively-thresholded Low Rank Approximation (ALRA) that deals with sparse matrices. Four tests available within the Seurat package were used to evaluate the differential expression: Wilcoxon test (Wilcox), Likelihood-ratio test for scRNASeq (bimod), Student’s t-test (t), and MAST.

### Statistical analysis

Data were presented as mean±SEM values of at least 3 independent experiments and analyzed by GraphPad Prism 7.0 software. Comparisons between groups were done by unpaired Student’s t-tests or Mann-Whitney´s test. p<0.05 was considered significant.

## Supporting information

S1 Fig Basal levels of dsDNA. (A) Basal raw numbers of dsDNA of (5 x 10^5^ cells/ well) of B/c (bluecircles) or B6 (purple circles) mice. Results presented as arbitrary units (AU) are mean±SEM of n = 6–24. Differences (*) between indicated bars were considered significant when p<0.05. Basal NET-DNA concentrations (mean ± SEM): 509.5±170 ng/mL (BMN B/c 2h), 441.8±123.5 ng/mL (BMN B6 2h), 507.6±74.53 ng/mL (BMN B/c 4h), 273.8±31.47 ng/mL (BMN B6 4h), 161.3±21.13 ng/mL (iNØ B/c 2h), 216.5±20.16 ng/mL (iNØ B6 2h), 281.1±50.69 ng/mL (iNØ B/c 4h), and 288.9±41.1 ng/mL (iNØ B6 4h). (B) BMN (BMN; 5 x 10^5^ cells/ well) of B/c (blue bars) or B6 (purple bars) mice were either left untreated (Nil) or infected with increasing doses of *L*. *major* promastigotes (indicated MOI) for 2 or 4 h and we quantified (B) elastase activity represented as arbitrary units.
(PDF)

S2 Fig NET release and inflammatory infiltrate elicited by *Leishmania major* in the peritoneum. (A) NET-DNA measured from peritoneal lavage fluid 3 h after i.p. injection of 10^6^ *L*. *major* metacyclic promastigotes inoculation in B/c (white circles) or B6 (gray circles) mice. (B) Percentage of Ly6G^+^ neutrophils from peritoneal lavage fluid. (C) Total number of neutrophils recovered after i.p. parasite injection. Results are mean±SEM of n = 6–7. # p<0.05.
(PDF)

S3 Fig Strain differences in NET formation are retained in highly purified neutrophils from a secondary animal facility and using an alternative quantification method. To certify the reproducibility of our data, we addressed NET formation in a secondary laboratory using a different methodology and animals obtained from another source (Taconic Biosciences). BMN obtained by negative selection with magnetic beads were stimulated with metacyclic promastigotes for 3 h, and extracellular dsDNA released in the supernatant was measured in a Nanodrop at 260 nm. Results are mean±SEM of n = 5–6. *p<0.05.
(PDF)

S4 Fig Production of NETs in response to *Leishmania sp*. is independent of TLR4 recognition. (A) BMN were stained with DAPI (blue) to reveal nucleus location and extracellular DNA of B6 wild-type (WT) untreated neutrophil (Naïve), or WT and TLR4^-/-^ treated with PMA (100 nM) or with *L*. *amazonensis* (1:5) promastigotes. (B) BMN (5 x 10^5^ cells/ well) of wild-type (black bars), or TLR4^-/-^ mice (yellow bars) were either left untreated (-) or incubated with increasing ratios of *L*. *major* promastigotes (1:1, 1:5) or *L*. *amazonensis* (La; 1:5) and NET generation quantified as extracellular dsDNA after 4 h. Results are mean±SEM of n = 2–3.
(PDF)

S5 Fig Human elastase penetrates the promastigote cell membrane. *L*. *major* WT (A, D), Δisp2/3 (B, E), and Δisp2/3: ISP2/3 (C, F) promastigotes were incubated with 10 μg ml^-1^ recombinant human neutrophil elastase (left panel) or in medium alone (right panel) for 2 h at 35°C and 5% CO_2_. Parasites were then fixed in 1% formaldehyde and left to adhere to poly-L-lysine coated slides. Parasites were permeabilized and stained with rabbit anti-neutrophil elastase pAb (1:200) followed by goat anti-rabbit Alexa Fluor 488-conjugated antibody (1:2000; Invitrogen). Slides were mounted with ProLong Gold antifade (Invitrogen). Images were obtained on a fluorescence microscope. Scale bar: 10 μm.
(PDF)

S6 Fig Knockout of ISP2/3 in *L*. *major* diminishes neutrophil phagocytosis. BMN (5 x 10^5^ cells/well) from B/c (white bars), B6 (black bars), and ELAKO (hatched bars) mice were incubated with CFSE-labeled *L*. *major* WT, Δisp2/3, and Δisp2/3: ISP2/3 promastigotes (1:5) for 4 h. Neutrophils were identified by anti-Ly6G-APC-Cy7 and further analyzed by staining with anti-CD11b-PE. Results show the percentage of CFSE^+^ neutrophils (CD11b^+^Ly6G^+^) are mean±SEM of n = 3 from 3 independent experiments. **p<0.01 and *** p<0.001.
(PDF)

S7 Fig MPO is associated with NET-DNA scaffold in neutrophils stimulated with parasites. _i_NØs (1 × 10^5^ cells) from B/c (A-C) and B6 (D-F) were incubated with *L*. *major* promastigotes (5 × 10^5^) for 4h at 35°C with 5% CO2 and fixed with 4% formaldehyde. Slides were stained with rabbit-polyclonal anti-MPO (Red; 1:50; Abcam) followed by goat-anti-rabbit-Alexa 546 (1:800; Molecular Probes) and mounted with ProLong Gold antifade reagent with DAPI (Blue; Thermo Fisher). White arrows point to MPO associated to NET fibers. Images were taken in a Leica DMI 6000 microscope. (C, F) Overlays.
(PDF)

S8 Fig Subcellular distribution of myeloperoxidase (MPO). BMN were stimulated or not with PMA/Ionomycin (80 nM/ 1.3 μM) at the indicated time points, e.g., 15-B (15 min, B/c neutrophils) or 15-C (15 min, B6 neutrophils). The intensity of anti-MPO Ab was analyzed in (A) cytosol or (B) nuclear fraction. Each circle represents one cell. UT = untreated neutrophils; BALB/c (B–white columns); C57BL6 (C- gray columns.) (C) dsDNA released by neutrophils from BALB/c (white columns) or C57BL/6 (gray columns) measured in a Nanodrop for the same time period as A, B. Results are mean±SEM of n = 4–6. *p<0.05. Compilation of 4–6 independent experiments.
(PDF)

S9 Fig Gene set enrichment analysis was performed on all probes using Gene Ontology annotations through the cluster Profiler. A ranked file containing gene names and log2 fold change values respective of all probes was used as input. (A) Multiple probes targeting the same gene were aggregated using the median of the probes. The significant pathways were selected based on an FDR < 0.001, white bars indicate ontologies enriched in the B/c naive in comparison with gray bars representing ontologies enriched in the B6 naive. (B) Gene set enrichment analysis as shown in (A) evidencing the number of genes matching the ontology (count) and the ratio of those genes considering all the genes in the ontology relative to B/c naïve samples. Activated ontologies indicate enrichment in the B/c naïve, whilst suppressed represent ontologies enriched in the B6 naive.
(PDF)

S10 Fig Frequency and relative number of intralesional neutrophil infiltration 4 weeks post-infection. BALB/c and C57BL/6 mice were infected with 10^6^ *L*. *major* stationary phase promastigotes i.d. and we evaluated the frequency of intralesional CD11b^+^Ly6G^+^ neutrophilic infiltrate (A) and (B) relative numbers of CD11b^+^Ly6G^+^ cells in mouse ears 4wks p.i. Results are mean±SEM of n = 4–5. Differences (*) were considered significant when p<0.05.
(PDF)

S11 Fig The transcriptional signature of neutrophil subpopulations regulates microbicidal response pathways. Neutrophil subsets (A) Top 10 marker genes of neutrophil subtypes identified with FindAllMarkers from Seurat v4. Genes that are more expressed are represented in yellow, and genes that are less expressed are represented in purple. Rows represent the top marker genes, and columns represent the expression of the cells from each neutrophil subtype. (B-C) Genes differentially expressed between N1 and N2 subtypes were identified using four statistical tests to increase the reliability and diminish limitations of differential expression in scRNASeq data. Results of those tests are shown for (B) phagocytosis-related genes and (C) NET release-related genes. Genes belonging to the signature below the threshold for differential expression were omitted. The axis depicts the average log2 fold change and the adjusted p-value identified by the tests. Genes are represented by colors, and the statistical tests are represented by different shapes, as shown in the legends.
(PDF)

S12 Fig Digestion and purification of Ly6G Fab fragments. Ly6G antibodies were cleaved with papain using Pierce Fab fragmentation kit following instructions provided by the manufacturer. (A) Fab fragments were purified using protein G agarose beads and a single 50 kDa band was obtained. (B) Purified Ly6G Fab was labeled with Alexa Fluor 647 and showed dose-dependent binding to splenocytes. (C) The specificity of Fab fragments was attested by the strong correlation with cells targeted by the whole IgG Ly6G antibody. Results are representative of 2 independent experiments.
(PDF)

S13 Fig Neutrophils form NETs within swarms at the early stages of *Leishmania amazonensis* infection. Mice were inoculated with CSFE-*L*. *amazonensis* (red) to achieve a small focal infection in the ear pinnae. BV421-conjugated anti-Ly6G antibody [blue] was i.v. injected 1 h later. Two hours after parasite inoculation, mice were anesthetized, injected with Sytox green i.v. and immediately imaged on a confocal microscope under 20X magnification. Time-lapse movies were created from sequential images taken every minute for 45 minutes. (A, B) Representative images from B6 (A) and BALB/c (B) mice, bottom panels are representative snapshots of two independent experiments. (C) Quantification of the Ly6G^+^ to Sytox^+^ area ratio for B6 and BALB/c mice was done at 15, 30, and 60 minutes. Results are mean±SEM of n = 3.
(PDF)

S14 Fig Ecto-3’-nucleotidase mediated NETs evasion promotes parasite invasion *in vivo*. *Leishmania infantum* 3’-nucleotidase activity was measured by 3’AMP hydrolysis as described on Material and Methods. LP parasites, as expected, showed greater 3’-nucleotidase activity than HP parasites (A). B/c mice were injected subcutaneously in the footpad with HP or LP parasites. After 26 days, parasite counts in the site of lesion (B) and draining lymph nodes (C) were analyzed by limiting dilution. Results are mean±SEM of n = 3.
(PNG)

S1 Video Tridimensional reconstruction of intranuclear MPO in neutrophils activated with PMA and ionomycin. (MP4)

S2 Video IVM showing swarming of neutrophils at the site of *L*. *amazonensis*-CSFE inoculation in the dermis. (MP4)

S3 Video IVM showing swarming of neutrophils and netosis at the site of *L*. *amazonensis*-RFP inoculation in the dermis. (MP4)

S1 Table A manually curated list of genes related to NET release (51 genes) or phagocytosis (65 genes) was obtained from established literature and used to select probes from the differential expression analysis. (PDF)
