# Supplementary figures and images for: Functional plasticity shapes neutrophil response to Leishmania major infection in susceptible and resistant strains of mice
Source: PLoS Pathog. 2024 Oct 8;20(10):e1012592. doi: 10.1371/journal.ppat.1012592 (PMC11488723; doi:10.1371/journal.ppat.1012592)

**Figure S1**

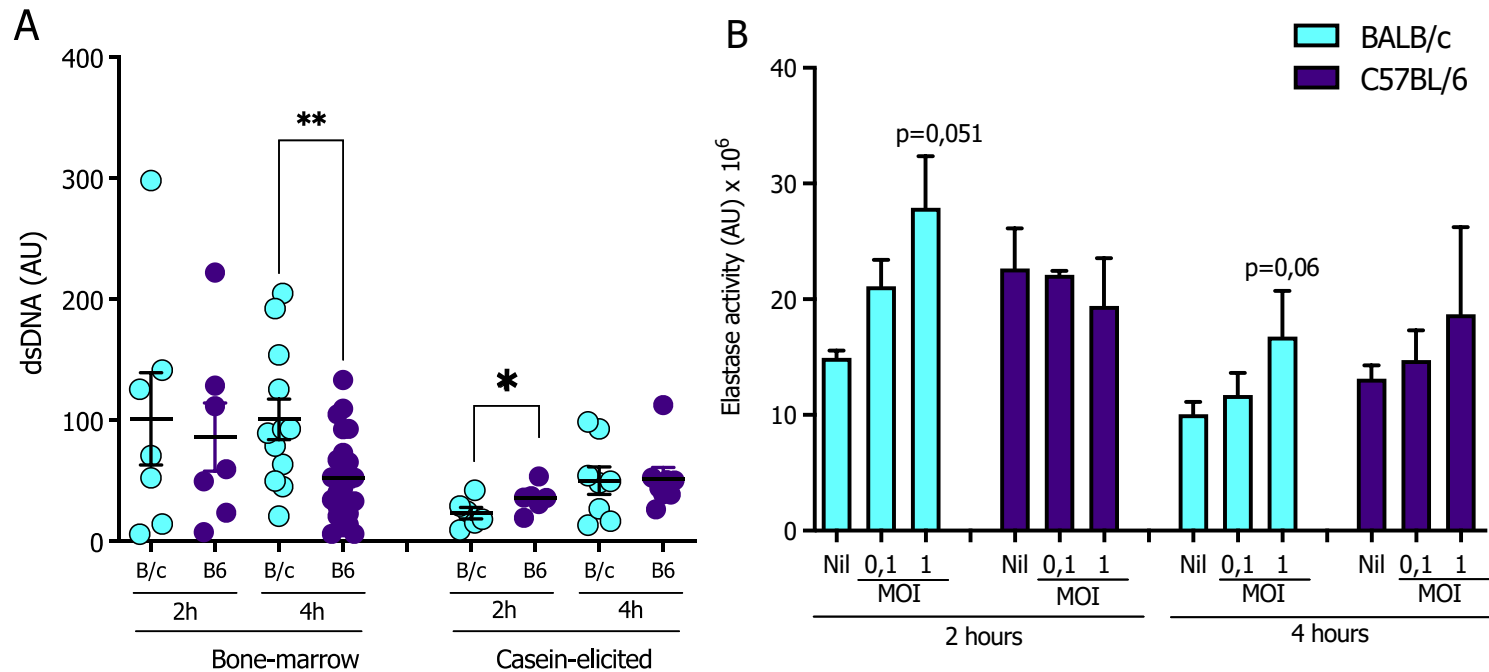

Supplement: S1 Fig — (A) Basal raw numbers of dsDNA of (5 x 10^5^ cells/ well) of B/c (bluecircles) or B6 (purple circles) mice. Results presented as arbitrary units (AU) are mean±SEM of n = 6–24. Differences (*) between indicated bars were considered significant when p<0.05. Basal NET-DNA concentrations (mean ± SEM): 509.5±170 ng/mL (BMN B/c 2h), 441.8±123.5 ng/mL (BMN B6 2h), 507.6±74.53 ng/mL (BMN B/c 4h), 273.8±31.47 ng/mL (BMN B6 4h), 161.3±21.13 ng/mL (iNØ B/c 2h), 216.5±20.16 ng/mL (iNØ B6 2h), 281.1±50.69 ng/mL (iNØ B/c 4h), and 288.9±41.1 ng/mL (iNØ B6 4h). (B) BMN (BMN; 5 x 10^5^ cells/ well) of B/c (blue bars) or B6 (purple bars) mice were either left untreated (Nil) or infected with increasing doses of L. major promastigotes (indicated MOI) for 2 or 4 h and we quantified (B) elastase activity represented as arbitrary units. (PDF) [file ppat.1012592.s001.pdf]

**Figure S2**

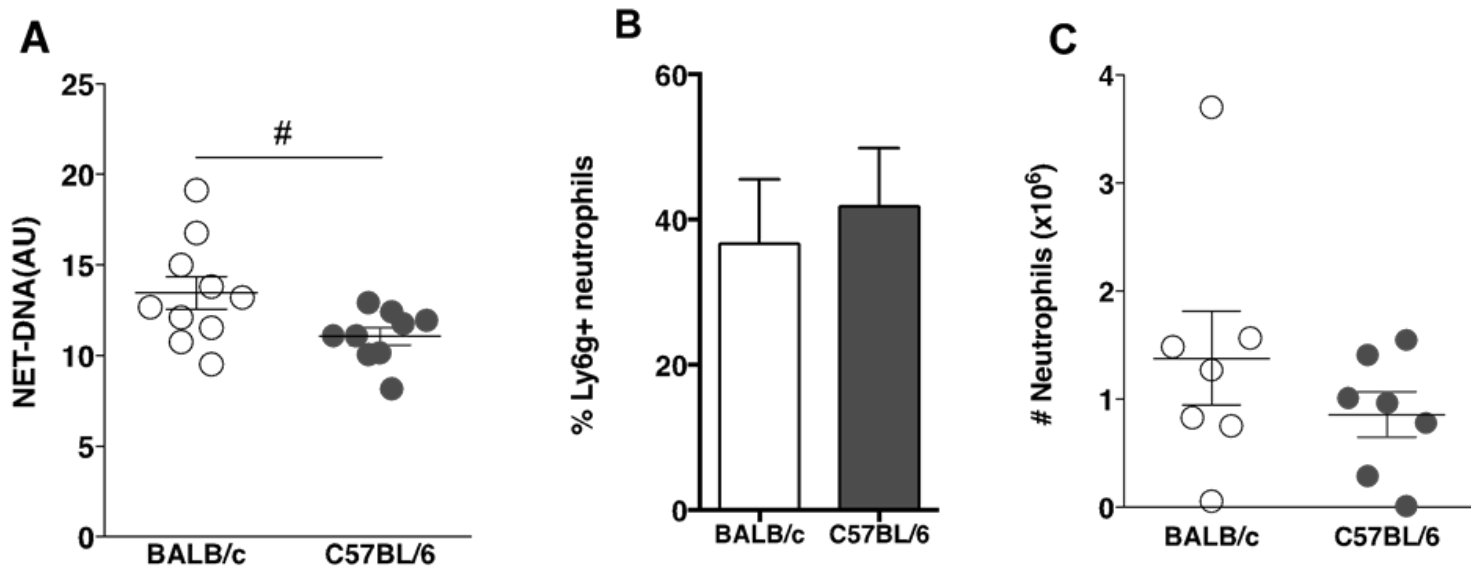

Supplement: S2 Fig — (A) NET-DNA measured from peritoneal lavage fluid 3 h after i.p. injection of 106 L. major metacyclic promastigotes inoculation in B/c (white circles) or B6 (gray circles) mice. (B) Percentage of Ly6G+ neutrophils from peritoneal lavage fluid. (C) Total number of neutrophils recovered after i.p. parasite injection. Results are mean±SEM of n = 6–7. # p<0.05. (PDF) [file ppat.1012592.s002.pdf]

Figure S3

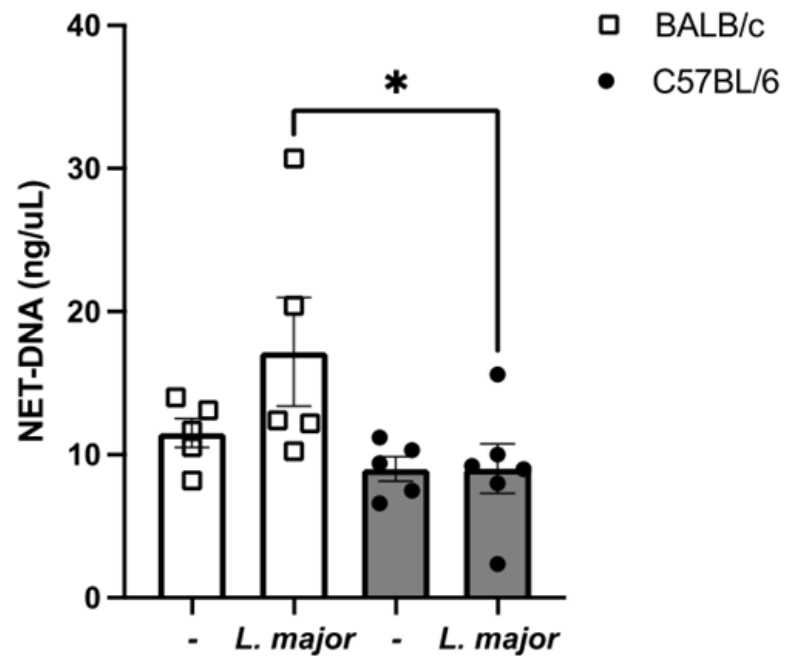

Supplement: S3 Fig — To certify the reproducibility of our data, we addressed NET formation in a secondary laboratory using a different methodology and animals obtained from another source (Taconic Biosciences). BMN obtained by negative selection with magnetic beads were stimulated with metacyclic promastigotes for 3 h, and extracellular dsDNA released in the supernatant was measured in a Nanodrop at 260 nm. Results are mean±SEM of n = 5–6. *p<0.05. (PDF) [file ppat.1012592.s003.pdf]

Figure S4

A

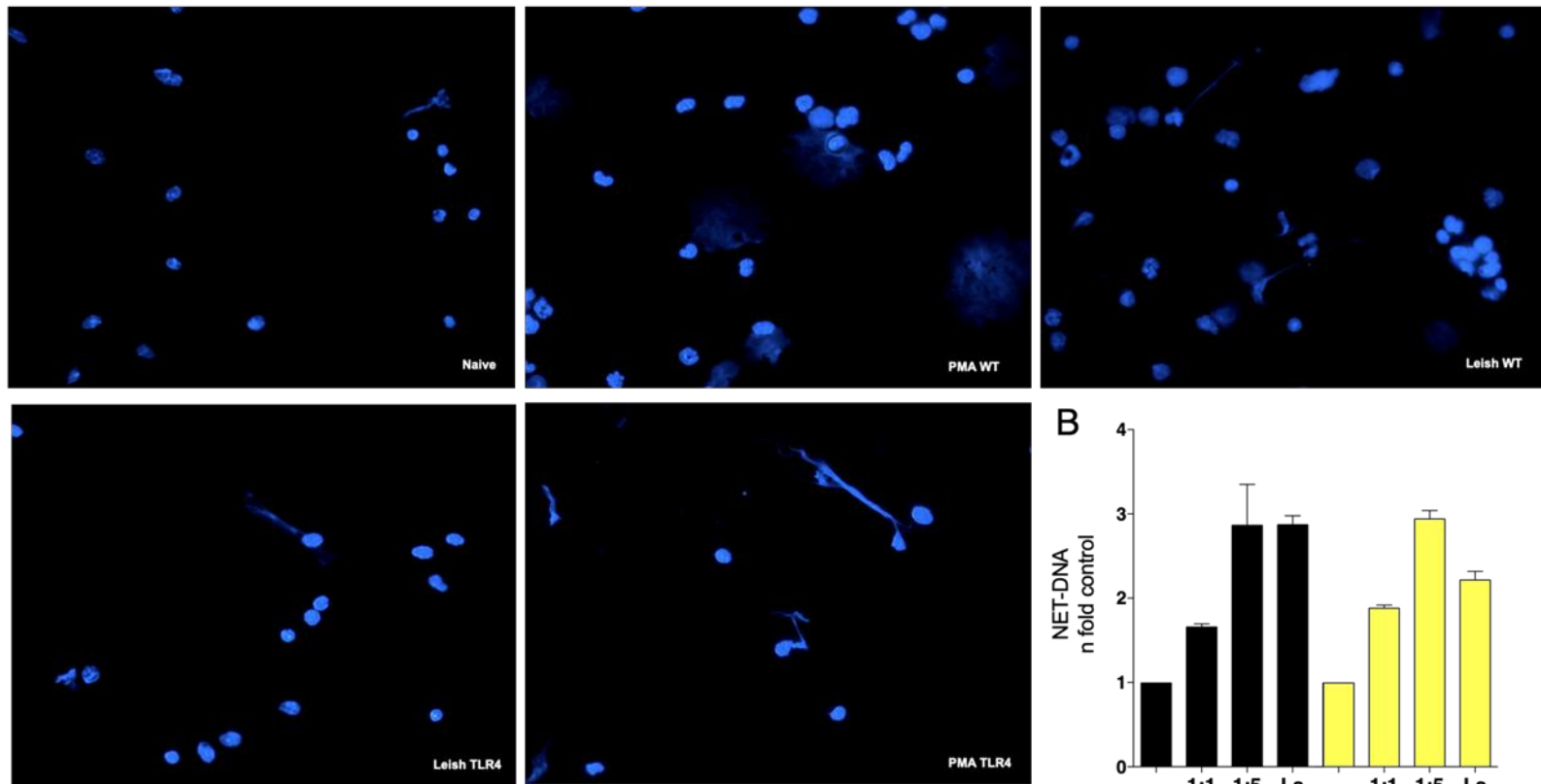

B

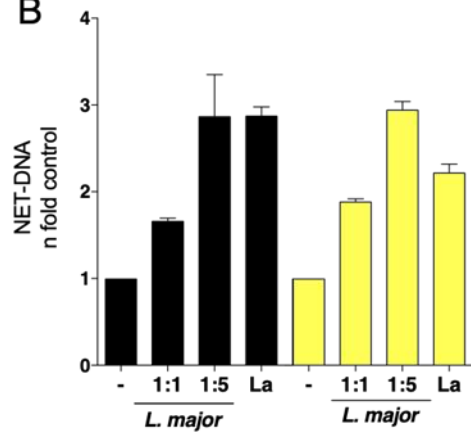

Supplement: S4 Fig — (A) BMN were stained with DAPI (blue) to reveal nucleus location and extracellular DNA of B6 wild-type (WT) untreated neutrophil (Naïve), or WT and TLR4-/- treated with PMA (100 nM) or with L. amazonensis (1:5) promastigotes. (B) BMN (5 x 10^5^ cells/ well) of wild-type (black bars), or TLR4-/- mice (yellow bars) were either left untreated (-) or incubated with increasing ratios of L. major promastigotes (1:1, 1:5) or L. amazonensis (La; 1:5) and NET generation quantified as extracellular dsDNA after 4 h. Results are mean±SEM of n = 2–3. (PDF) [file ppat.1012592.s004.pdf]

**Figure S5**

**+ Neutrophil elastase**

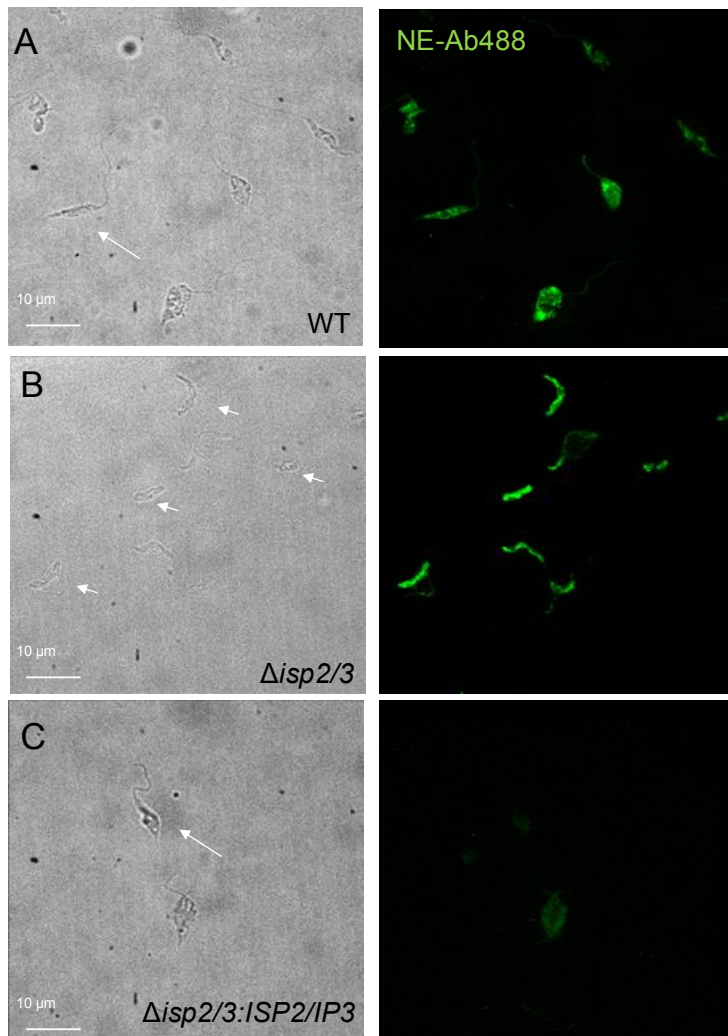

**- Neutrophil elastase**

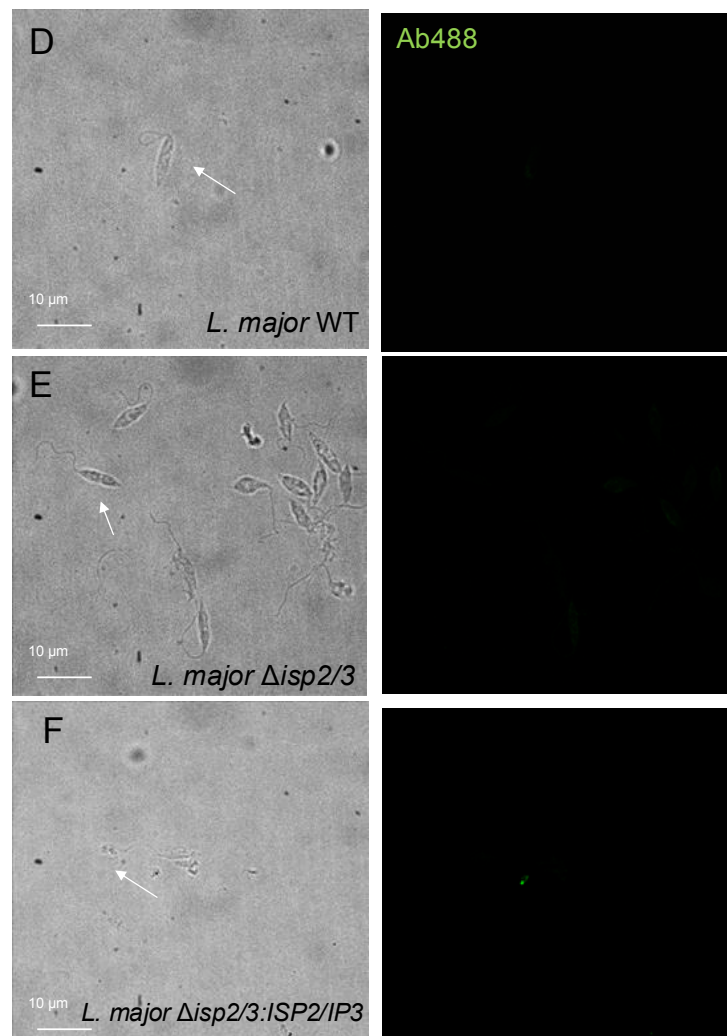

Supplement: S5 Fig — L. major WT (A, D), Δisp2/3 (B, E), and Δisp2/3: ISP2/3 (C, F) promastigotes were incubated with 10 μg ml-1 recombinant human neutrophil elastase (left panel) or in medium alone (right panel) for 2 h at 35°C and 5% CO2. Parasites were then fixed in 1% formaldehyde and left to adhere to poly-L-lysine coated slides. Parasites were permeabilized and stained with rabbit anti-neutrophil elastase pAb (1:200) followed by goat anti-rabbit Alexa Fluor 488-conjugated antibody (1:2000; Invitrogen). Slides were mounted with ProLong Gold antifade (Invitrogen). Images were obtained on a fluorescence microscope. Scale bar: 10 μm. (PDF) [file ppat.1012592.s005.pdf]

Figure S6

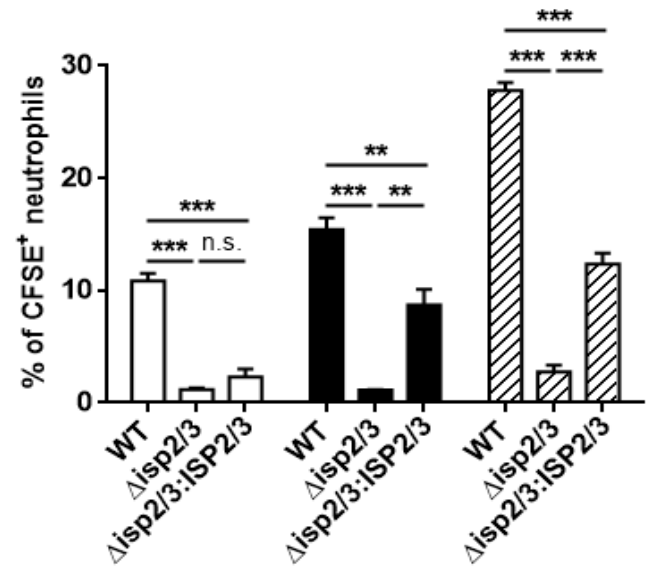

Supplement: S6 Fig — BMN (5 x 10^5^ cells/well) from B/c (white bars), B6 (black bars), and ELAKO (hatched bars) mice were incubated with CFSE-labeled L. major WT, Δisp2/3, and Δisp2/3: ISP2/3 promastigotes (1:5) for 4 h. Neutrophils were identified by anti-Ly6G-APC-Cy7 and further analyzed by staining with anti-CD11b-PE. Results show the percentage of CFSE+ neutrophils (CD11b+Ly6G+) are mean±SEM of n = 3 from 3 independent experiments. **p<0.01 and *** p<0.001. (PDF) [file ppat.1012592.s006.pdf]

Figure S7

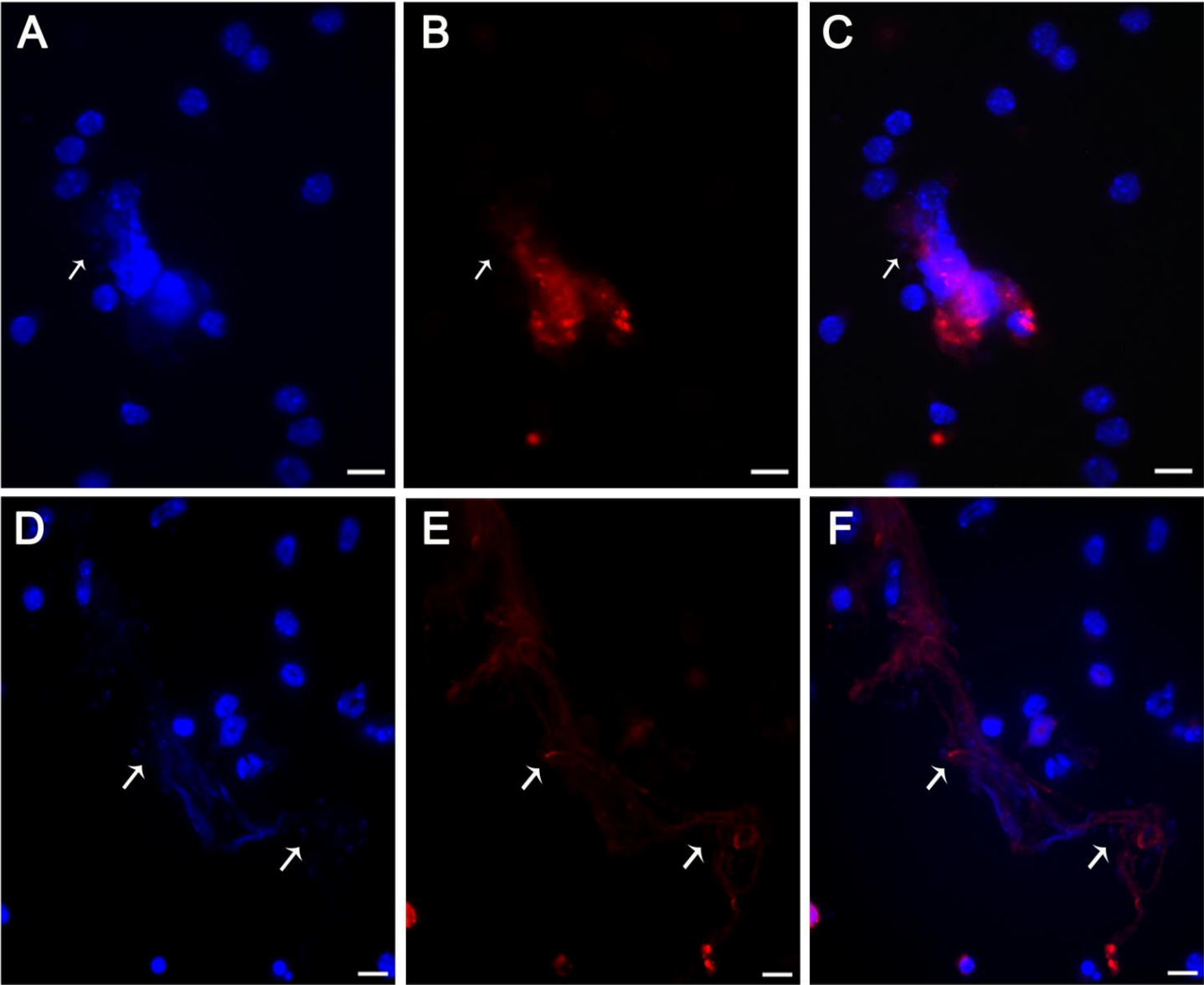

Supplement: S7 Fig — iNØs (1 × 10^5^ cells) from B/c (A-C) and B6 (D-F) were incubated with L. major promastigotes (5 × 10^5^) for 4h at 35°C with 5% CO2 and fixed with 4% formaldehyde. Slides were stained with rabbit-polyclonal anti-MPO (Red; 1:50; Abcam) followed by goat-anti-rabbit-Alexa 546 (1:800; Molecular Probes) and mounted with ProLong Gold antifade reagent with DAPI (Blue; Thermo Fisher). White arrows point to MPO associated to NET fibers. Images were taken in a Leica DMI 6000 microscope. (C, F) Overlays. (PDF) [file ppat.1012592.s007.pdf]

Figure S8

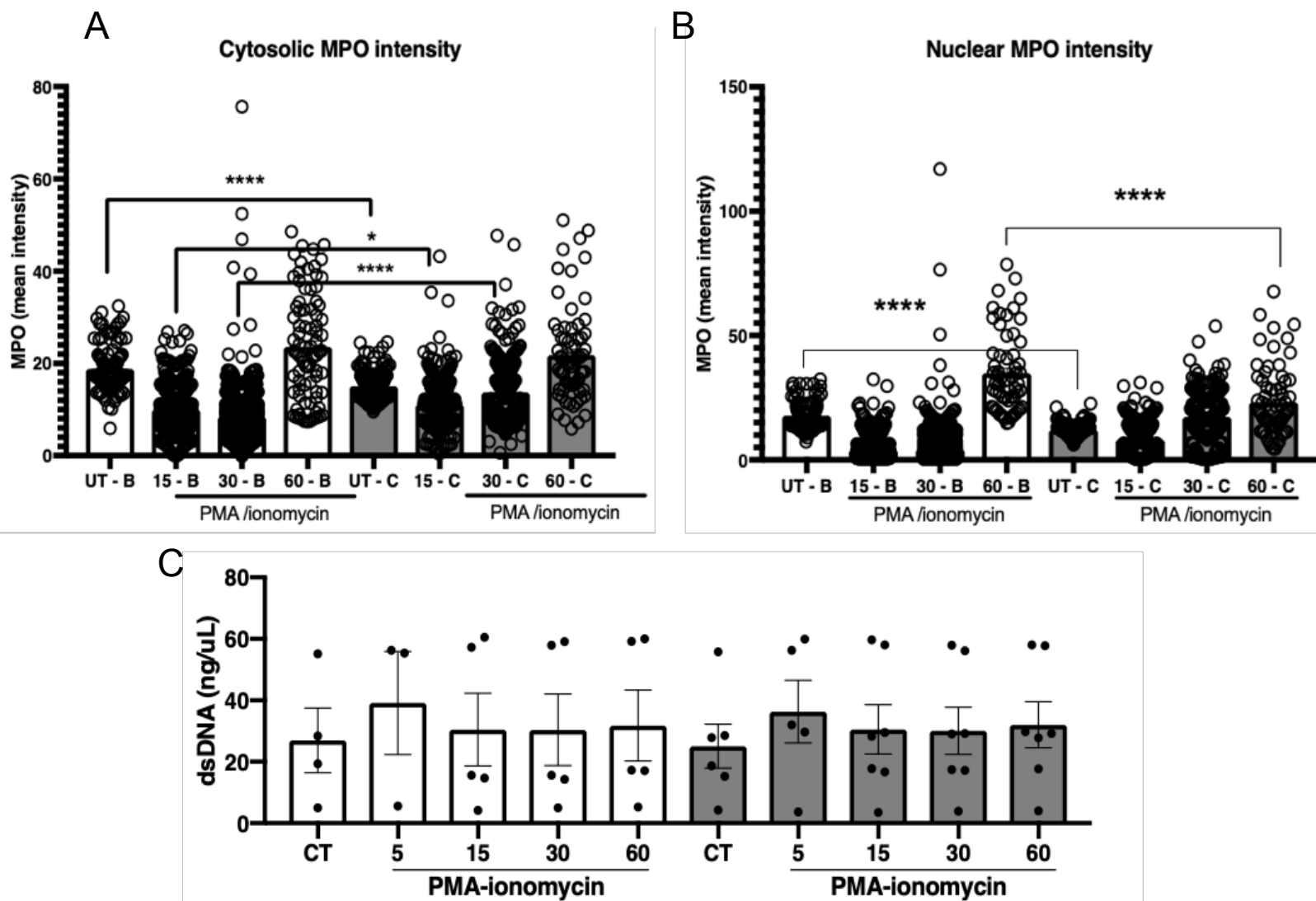

Supplement: S8 Fig — BMN were stimulated or not with PMA/Ionomycin (80 nM/ 1.3 μM) at the indicated time points, e.g., 15-B (15 min, B/c neutrophils) or 15-C (15 min, B6 neutrophils). The intensity of anti-MPO Ab was analyzed in (A) cytosol or (B) nuclear fraction. Each circle represents one cell. UT = untreated neutrophils; BALB/c (B–white columns); C57BL6 (C- gray columns.) (C) dsDNA released by neutrophils from BALB/c (white columns) or C57BL/6 (gray columns) measured in a Nanodrop for the same time period as A, B. Results are mean±SEM of n = 4–6. *p<0.05. Compilation of 4–6 independent experiments. (PDF) [file ppat.1012592.s008.pdf]

Figure S9

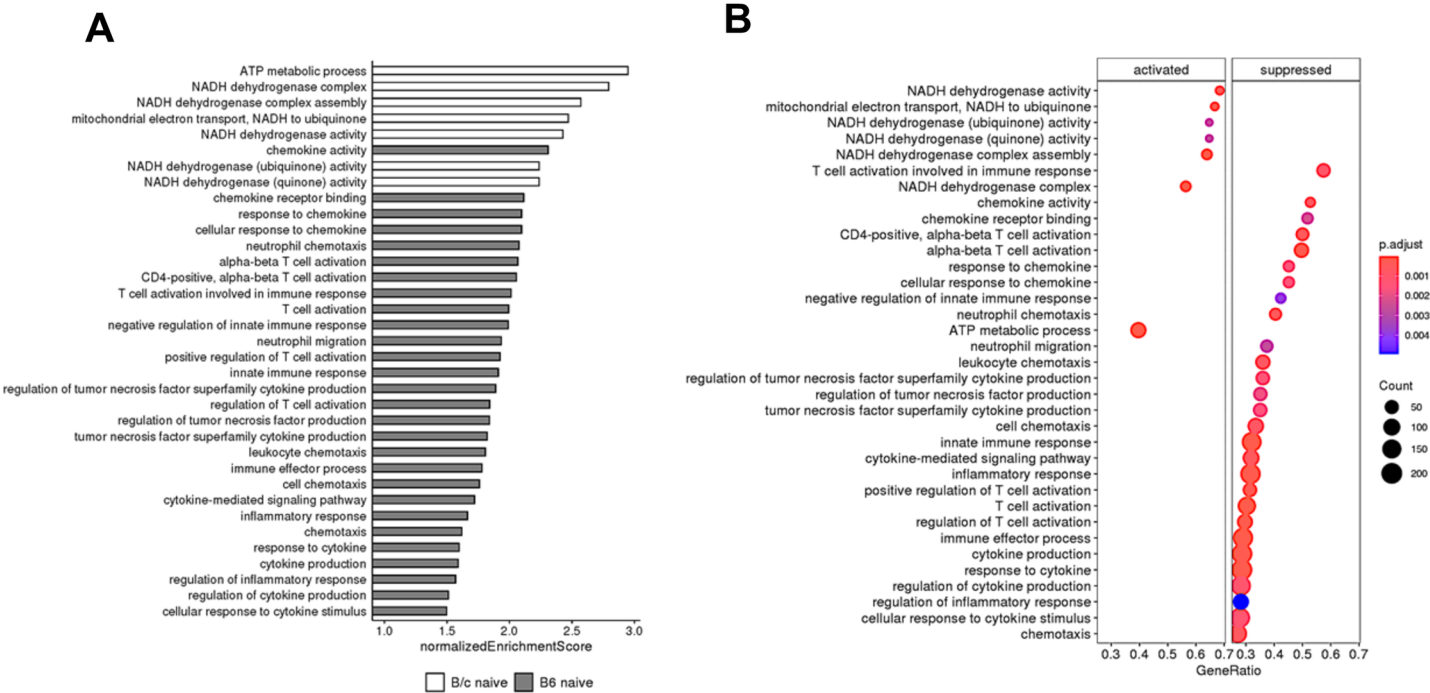

Supplement: S9 Fig — A ranked file containing gene names and log2 fold change values respective of all probes was used as input. (A) Multiple probes targeting the same gene were aggregated using the median of the probes. The significant pathways were selected based on an FDR < 0.001, white bars indicate ontologies enriched in the B/c naive in comparison with gray bars representing ontologies enriched in the B6 naive. (B) Gene set enrichment analysis as shown in (A) evidencing the number of genes matching the ontology (count) and the ratio of those genes considering all the genes in the ontology relative to B/c naïve samples. Activated ontologies indicate enrichment in the B/c naïve, whilst suppressed represent ontologies enriched in the B6 naive. (PDF) [file ppat.1012592.s009.pdf]

Figure S10

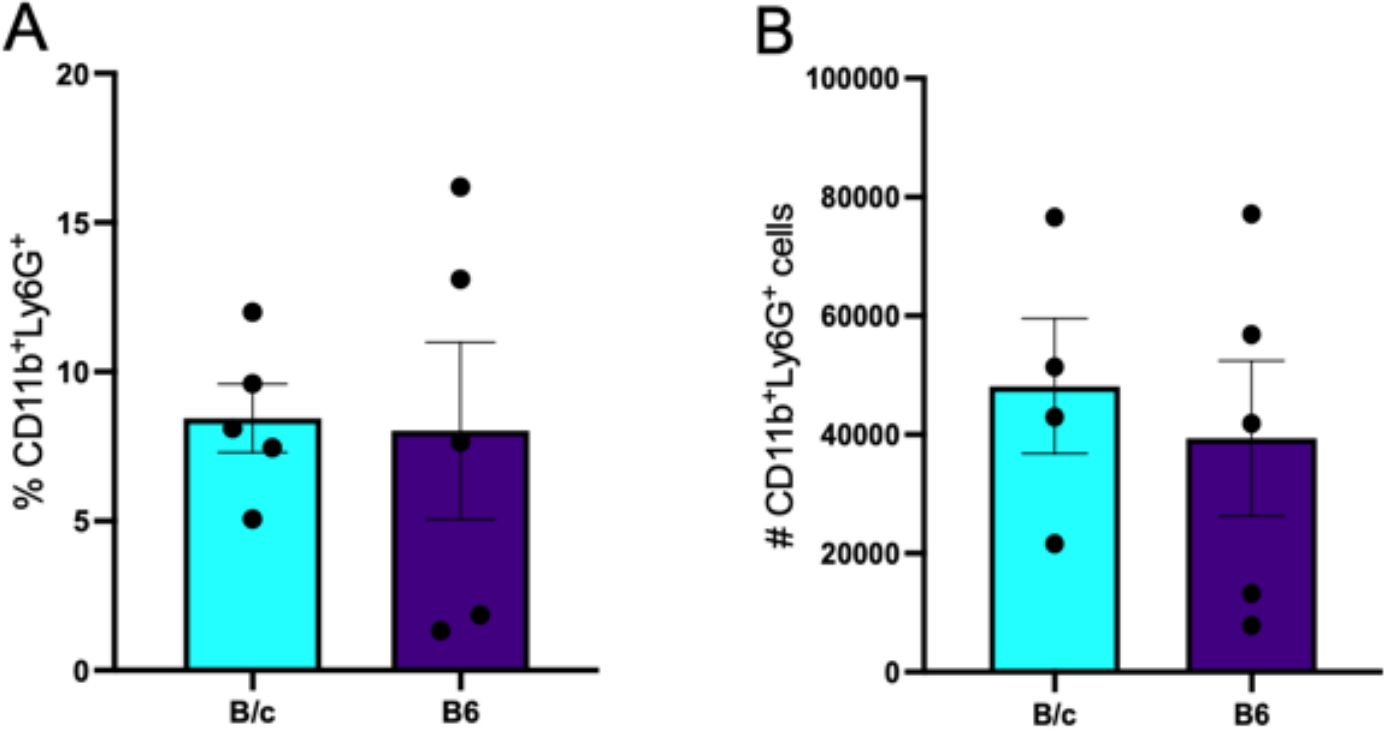

Supplement: S10 Fig — BALB/c and C57BL/6 mice were infected with 106 L. major stationary phase promastigotes i.d. and we evaluated the frequency of intralesional CD11b+Ly6G+ neutrophilic infiltrate (A) and (B) relative numbers of CD11b+Ly6G+ cells in mouse ears 4wks p.i. Results are mean±SEM of n = 4–5. Differences (*) were considered significant when p<0.05. (PDF) [file ppat.1012592.s010.pdf]

Figure S11

A

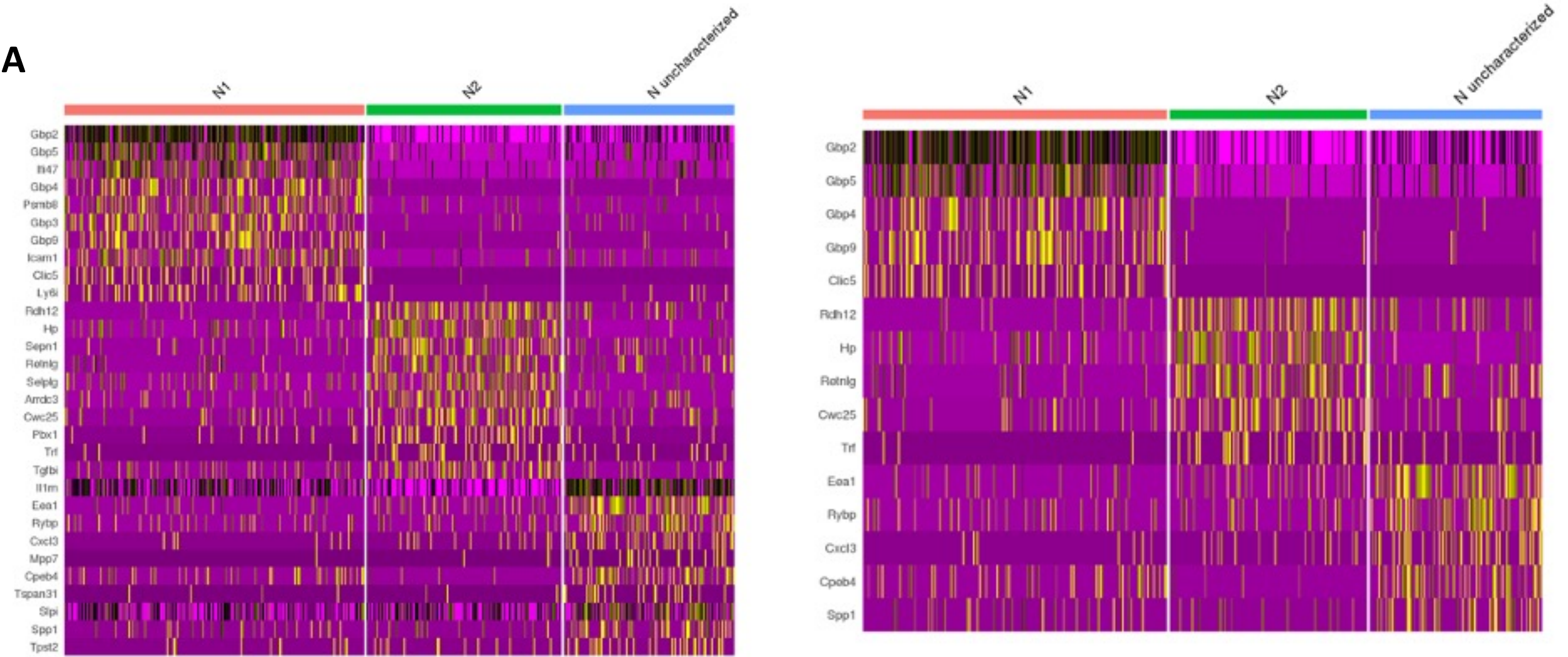

B

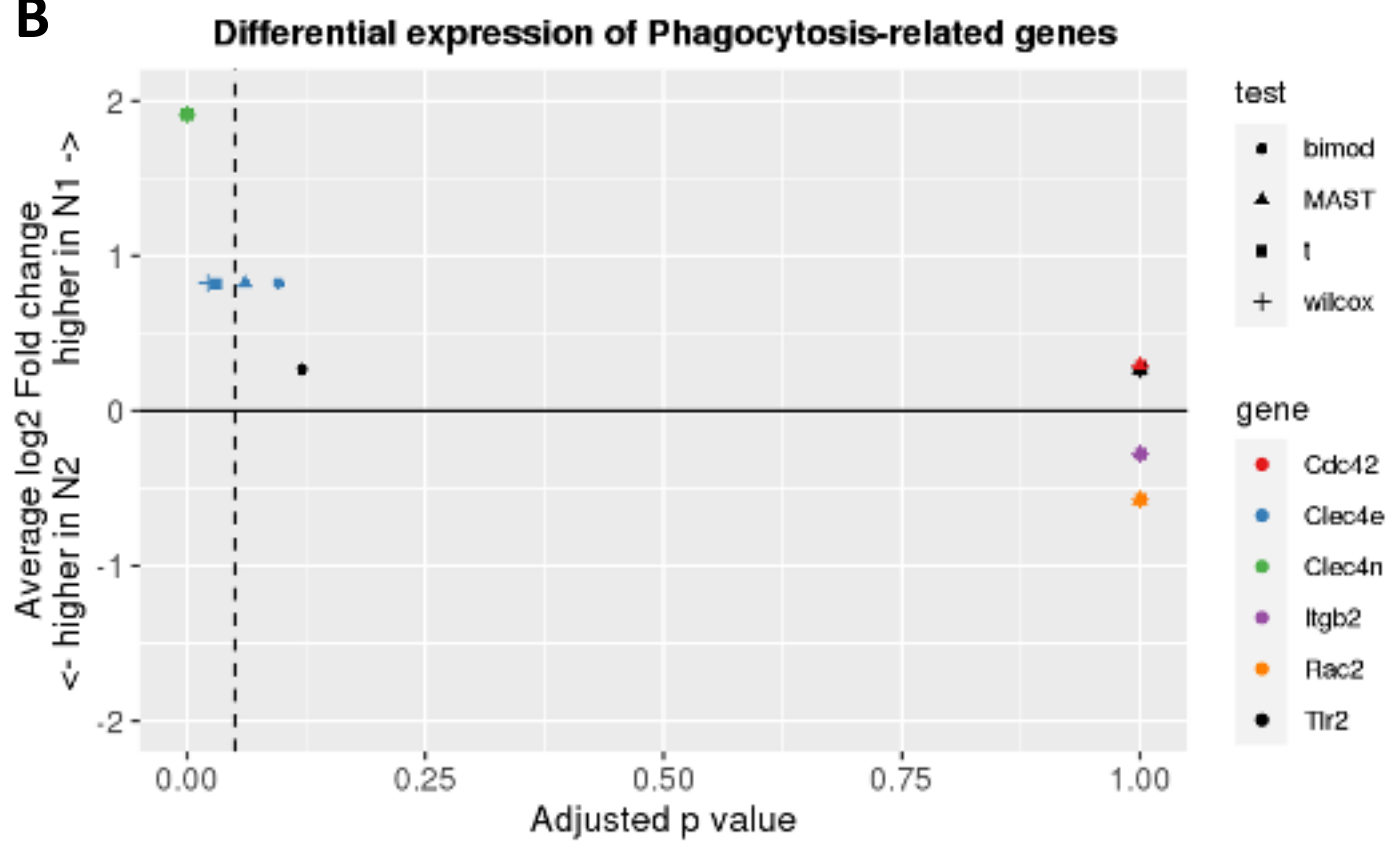

C

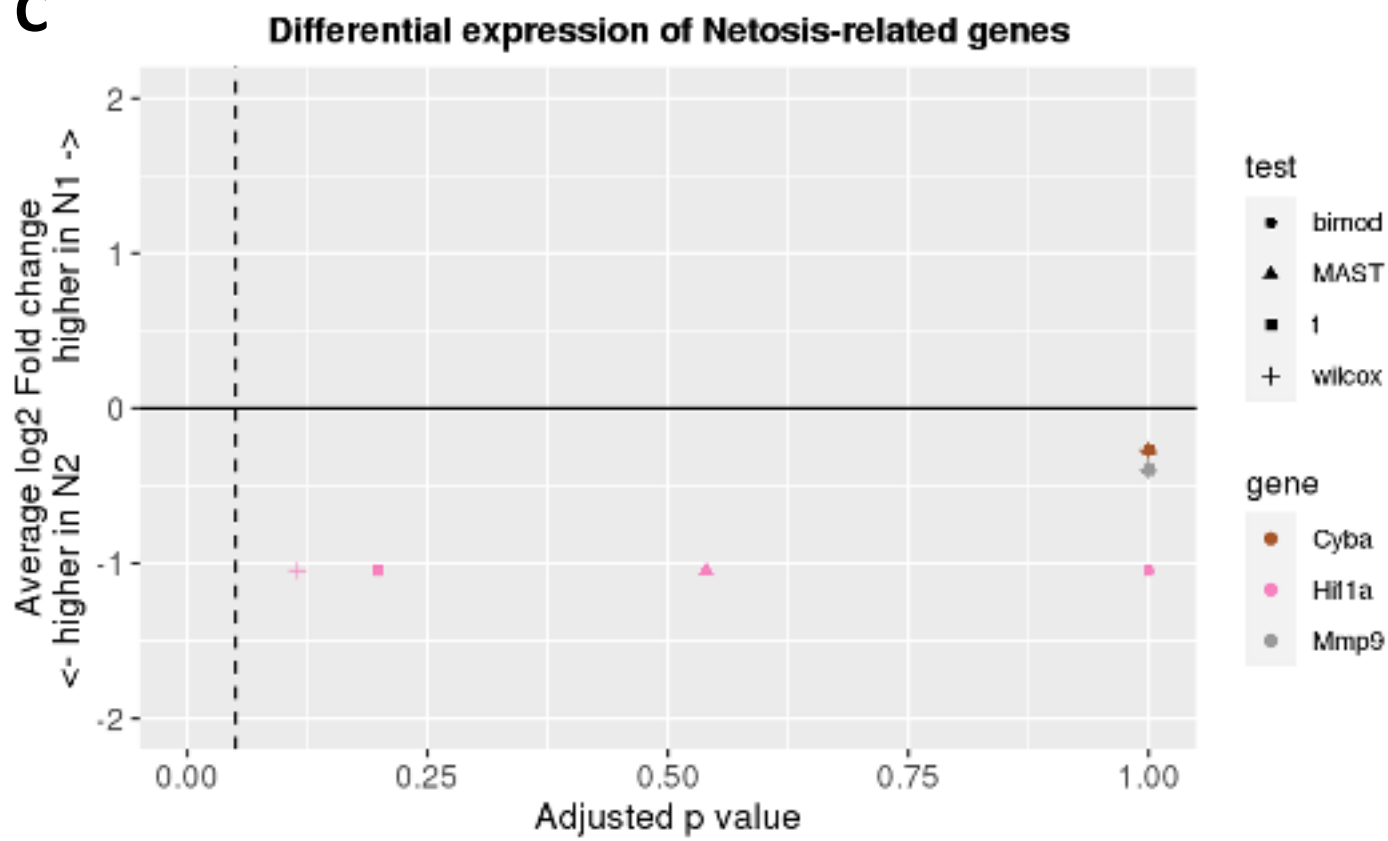

Supplement: S11 Fig — Neutrophil subsets (A) Top 10 marker genes of neutrophil subtypes identified with FindAllMarkers from Seurat v4. Genes that are more expressed are represented in yellow, and genes that are less expressed are represented in purple. Rows represent the top marker genes, and columns represent the expression of the cells from each neutrophil subtype. (B-C) Genes differentially expressed between N1 and N2 subtypes were identified using four statistical tests to increase the reliability and diminish limitations of differential expression in scRNASeq data. Results of those tests are shown for (B) phagocytosis-related genes and (C) NET release-related genes. Genes belonging to the signature below the threshold for differential expression were omitted. The axis depicts the average log2 fold change and the adjusted p-value identified by the tests. Genes are represented by colors, and the statistical tests are represented by different shapes, as shown in the legends. (PDF) [file ppat.1012592.s011.pdf]

Figure S12

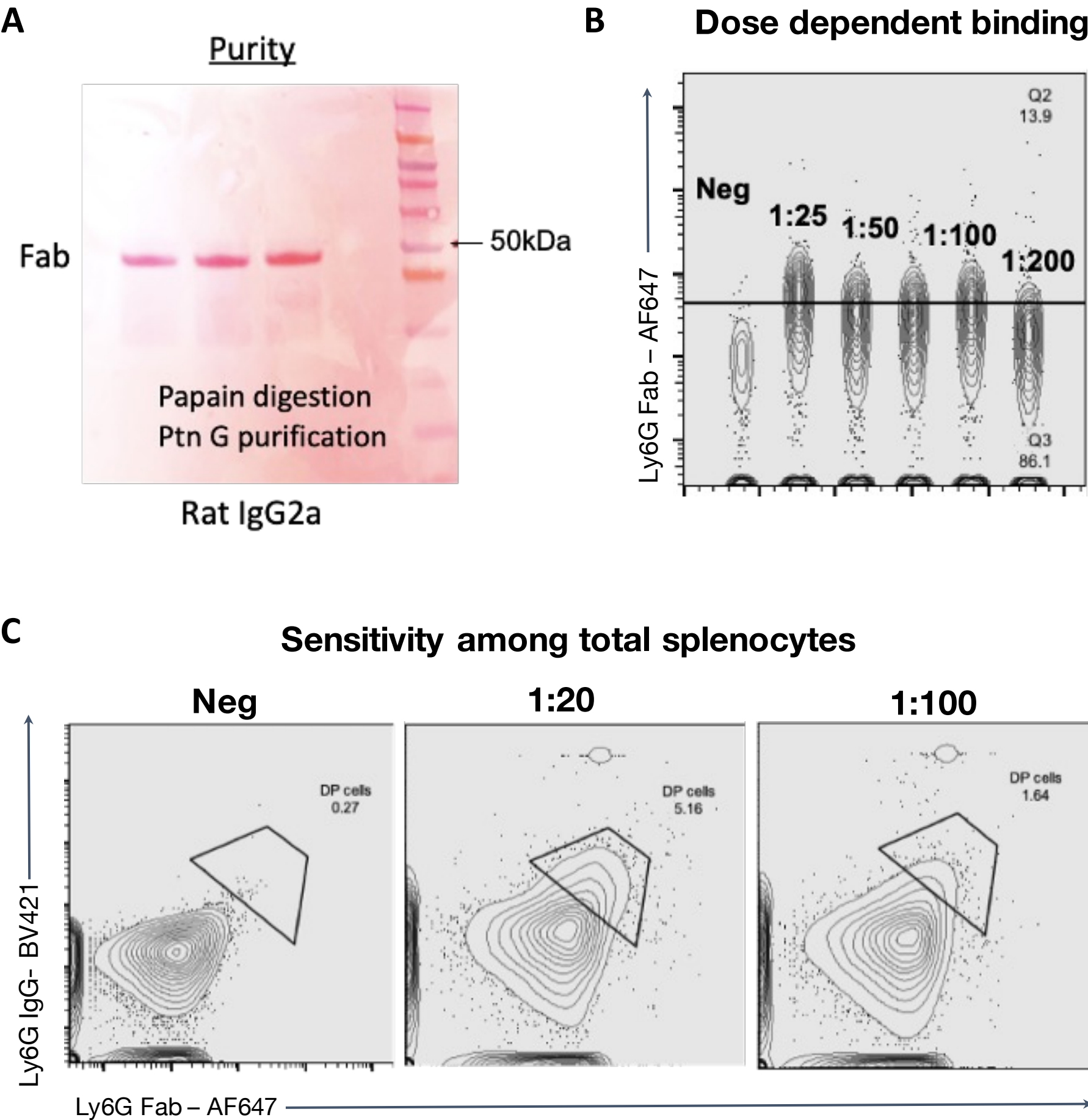

Supplement: S12 Fig — Ly6G antibodies were cleaved with papain using Pierce Fab fragmentation kit following instructions provided by the manufacturer. (A) Fab fragments were purified using protein G agarose beads and a single 50 kDa band was obtained. (B) Purified Ly6G Fab was labeled with Alexa Fluor 647 and showed dose-dependent binding to splenocytes. (C) The specificity of Fab fragments was attested by the strong correlation with cells targeted by the whole IgG Ly6G antibody. Results are representative of 2 independent experiments. (PDF) [file ppat.1012592.s012.pdf]

Figure S13

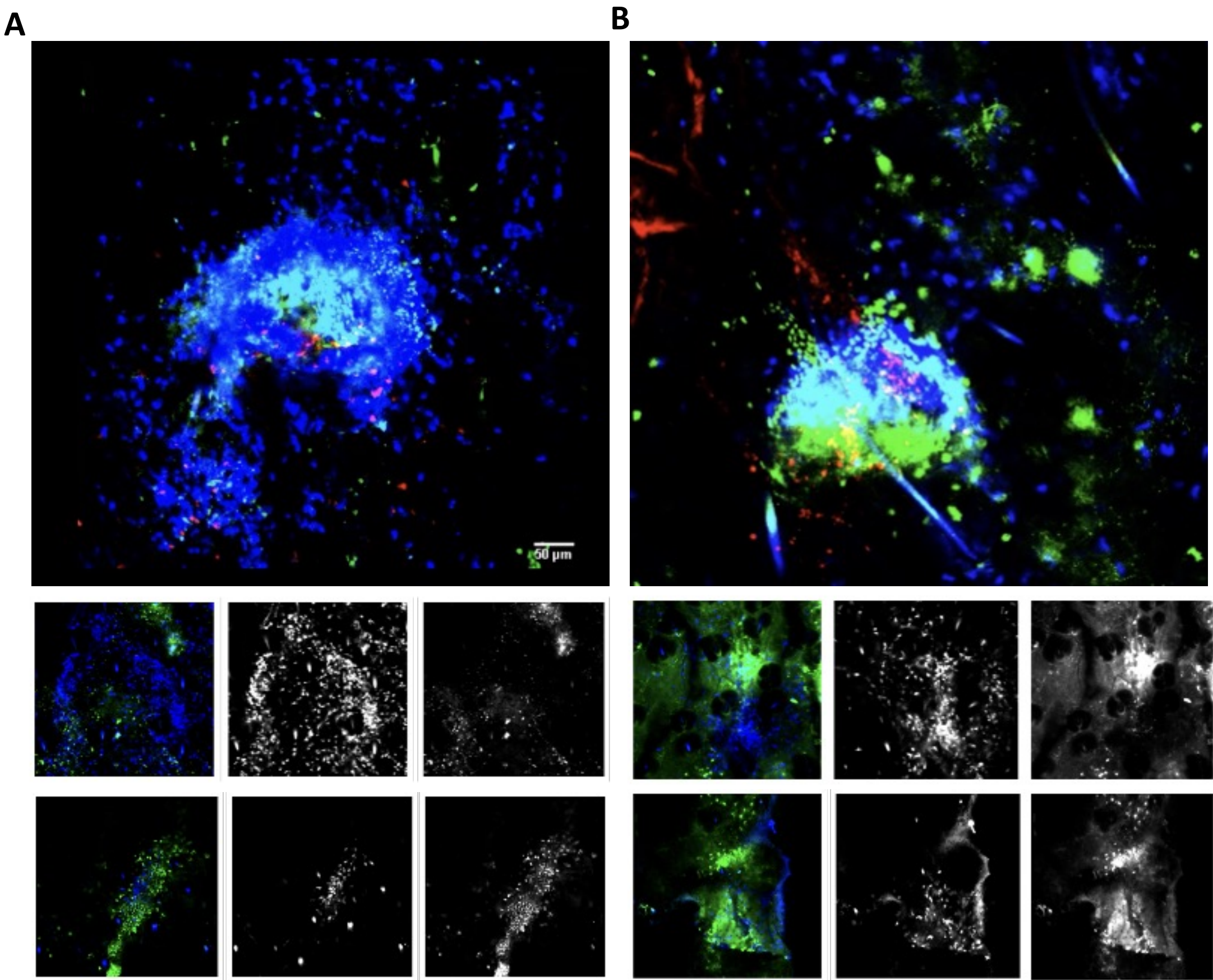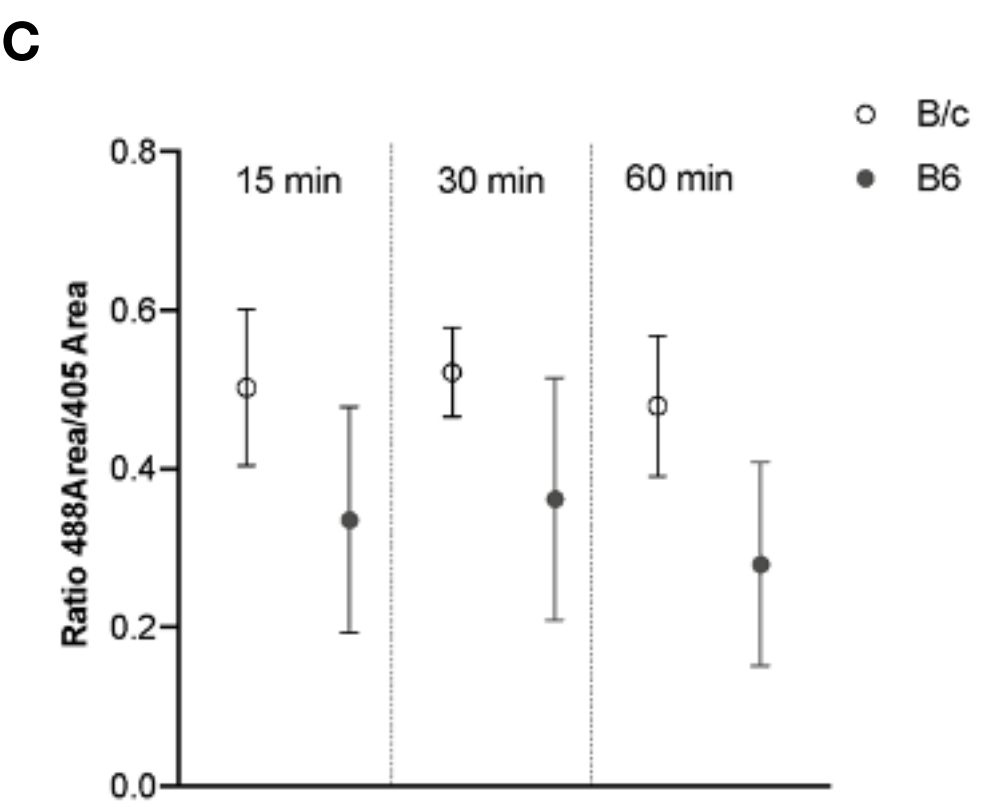

Supplement: S13 Fig — Mice were inoculated with CSFE-L. amazonensis (red) to achieve a small focal infection in the ear pinnae. BV421-conjugated anti-Ly6G antibody [blue] was i.v. injected 1 h later. Two hours after parasite inoculation, mice were anesthetized, injected with Sytox green i.v. and immediately imaged on a confocal microscope under 20X magnification. Time-lapse movies were created from sequential images taken every minute for 45 minutes. (A, B) Representative images from B6 (A) and BALB/c (B) mice, bottom panels are representative snapshots of two independent experiments. (C) Quantification of the Ly6G+ to Sytox+ area ratio for B6 and BALB/c mice was done at 15, 30, and 60 minutes. Results are mean±SEM of n = 3. (PDF) [file ppat.1012592.s013.pdf]

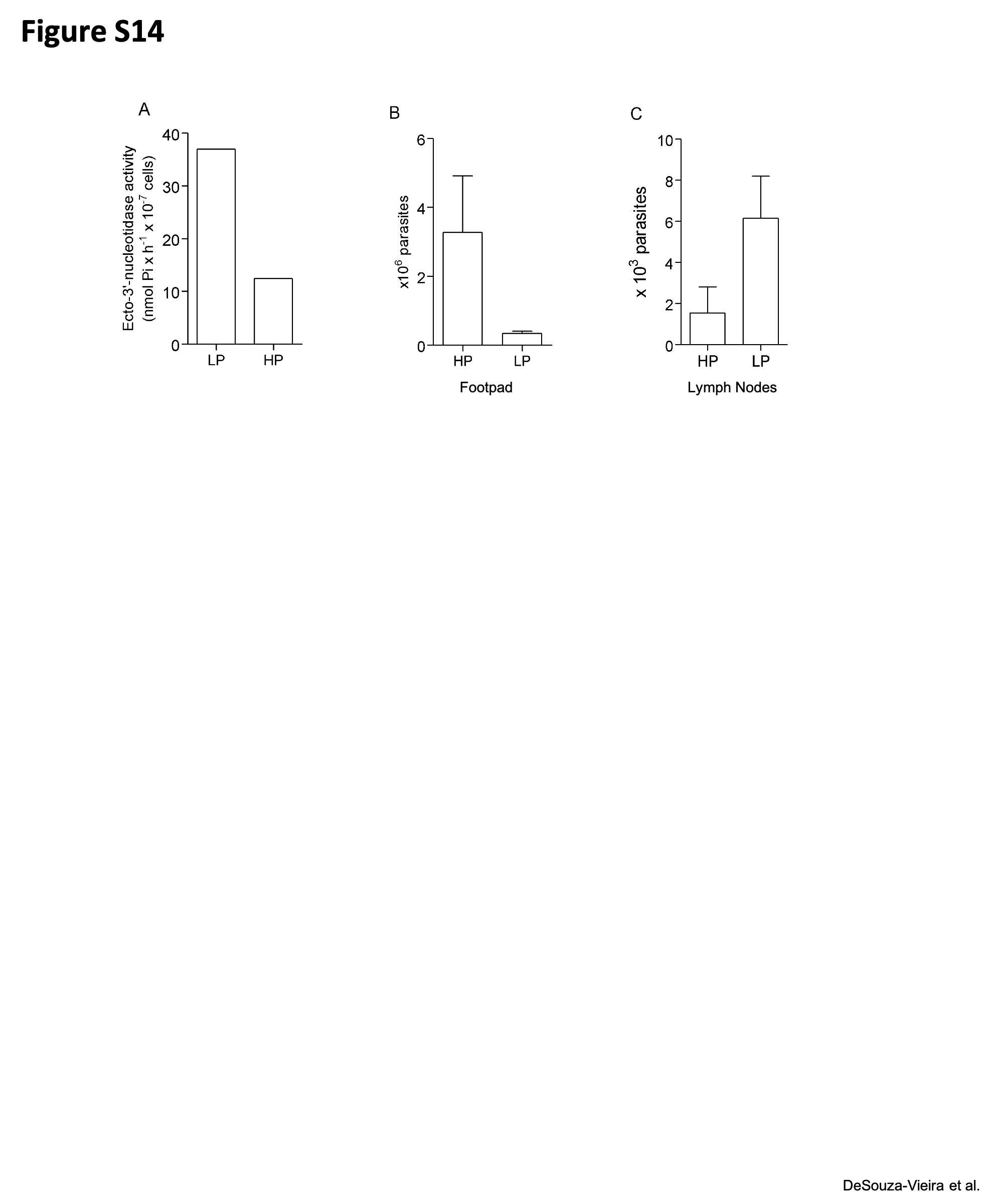

Supplement: S14 Fig — Leishmania infantum 3’-nucleotidase activity was measured by 3’AMP hydrolysis as described on Material and Methods. LP parasites, as expected, showed greater 3’-nucleotidase activity than HP parasites (A). B/c mice were injected subcutaneously in the footpad with HP or LP parasites. After 26 days, parasite counts in the site of lesion (B) and draining lymph nodes (C) were analyzed by limiting dilution. Results are mean±SEM of n = 3. (PNG) [file ppat.1012592.s014.png]
