# Supplementary material for: Functional plasticity shapes neutrophil response to Leishmania major infection in susceptible and resistant strains of mice
Source: PLoS Pathog. 2024 Oct 8;20(10):e1012592. doi: 10.1371/journal.ppat.1012592 (PMC11488723; doi:10.1371/journal.ppat.1012592)
Supplement: S1 Table — (PDF) [file ppat.1012592.s018.pdf]

Table S1

| List of manually curated genes related to neutrophils extracellular traps formation or phagocytosis | | |
|---|---|---|
| **Symbol.human** | **Symbol.mouse** | **Interest** |
| PADI4 | Padi4 | NET |
| ELANE | Elane | NET |
| MPO | Mpo | NET |
| CYBB | Cybb | NET |
| CYBA | Cyba | NET |
| NOX1 | Nox1 | NET |
| NOX3 | Nox3 | NET |
| NOX4 | Nox4 | NET |
| NOX5 | NA | NET |
| NOXO1 | Noxo1 | NET |
| NOXA1 | Noxa1 | NET |
| PRKCA | Prkca | NET |
| PRKCG | Prkcg | NET |
| PRKCB | Prkcb | NET |
| PRKCZ | Prkcz | NET |
| PRKCC | NA | NET |
| PRKCE | Prkce | NET |
| PRKCD | Prkcd | NET |

| | | |
|---|---|---|
| PIK3CG | Pik3cg | NET |
| PIK3CD | Pik3cd | NET |
| MAPK1 | Mapk1 | NET |
| MAP2K1 | Map2k1 | NET |
| MAPK3 | Mapk3 | NET |
| DNASE1 | Dnase1 | NET |
| GSDMD | Gsdmd | NET |
| GSDME | Gsdme | NET |
| GSDMB | NA | NET |
| GSDMA | Gsdma | NET |
| GSDMA | Gsdma2 | NET |
| GSDMA | Gsdma2 | NET |
| GSDMC | Gsdmc4 | NET |
| GSDMC | Gsdmc | NET |
| GSDMC | Gsdmc3 | NET |
| GSDMC | Gsdmc2 | NET |
| MMP9 | Mmp9 | NET |
| MMP2 | Mmp2 | NET |
| MMP1 | Mmp1a | NET |
| MMP14 | Mmp14 | NET |
| MMP3 | Mmp3 | NET |

| | | |
|---|---|---|
| MMP7 | Mmp7 | NET |
| MMP12 | Mmp12 | NET |
| MMP8 | Mmp8 | NET |
| MMP10 | Mmp10 | NET |
| MMP15 | Mmp15 | NET |
| TIMP2 | Timp2 | NET |
| TIMP1 | Timp1 | NET |
| TIMP3 | Timp3 | NET |
| CLEC7A | Clec7a | Phagocytosis |
| CLEC6A | Clec4n | Phagocytosis |
| TLR2 | Tlr2 | Phagocytosis |
| TLR4 | Tlr4 | Phagocytosis |
| ITGAM | Itgam | Phagocytosis |
| ITGB2 | Itgb2 | Phagocytosis |
| CLEC12A | Clec12a | Phagocytosis |
| CLEC4E | Clec4e | Phagocytosis |
| CLEC9A | Clec9a | Phagocytosis |
| MSR1 | Msr1 | Phagocytosis |
| MARCO | Marco | Phagocytosis |
| CD36 | Cd36 | Phagocytosis |
| CD33 | NA | Phagocytosis |

| | | |
|---|---|---|
| SIGLEC1 | Siglec1 | Phagocytosis |
| MTOR | Mtor | NET |
| PRKAR1A | Prkar1a | NET |
| PRKAR1B | Prkar1b | NET |
| PRKAR2A | Prkar2a | NET |
| PRKAR2B | Prkar2b | NET |
| CXCL2 | Cxcl1 | NET |
| HIF1A | Hif1a | NET |
| CD63 | Cd63 | Phagocytosis |
| CEACAM8 | Ceacam10 | Phagocytosis |
| CEACAM8 | Ceacam2 | Phagocytosis |
| CEACAM8 | Ceacam10 | Phagocytosis |
| ACTR2 | Actr2 | Phagocytosis |
| WAS | Was | Phagocytosis |
| WASL | Wipf1 | Phagocytosis |
| WIPF1 | Washc1 | Phagocytosis |
| WASHC1 | Washc1 | Phagocytosis |
| WASHC4 | Washc4 | Phagocytosis |
| WASHC5 | Washc5 | Phagocytosis |
| WASHC5C | | Phagocytosis |
| WASHC2A | Washc2 | Phagocytosis |

| | | |
|---|---|---|
| WASHC3 | Washc3 | Phagocytosis |
| WHAMM | Whamm | Phagocytosis |
| JMY | Jmy | Phagocytosis |
| VAV1 | Vav1 | Phagocytosis |
| VAV2 | Vav2 | Phagocytosis |
| VAV3 | Vav3 | Phagocytosis |
| ARF6 | Arf6 | Phagocytosis |
| VAMP3 | Vamp3 | Phagocytosis |
| CDC42 | Cdc42 | Phagocytosis |
| RHO | Rho | Phagocytosis |
| RAC1 | Rac1 | Phagocytosis |
| RAC2 | Rac2 | Phagocytosis |
| TRIM23 | Trim23 | Phagocytosis |
| ARF1 | Arf1 | Phagocytosis |
| ARF3 | Arf3 | Phagocytosis |
| ARF4 | Arf4 | Phagocytosis |
| ARF5 | Arf5 | Phagocytosis |
| ARF6 | Arf6 | Phagocytosis |
| ARL1 | Arl1 | Phagocytosis |
| ARL2 | Arl2 | Phagocytosis |
| ARL3 | Arl3 | Phagocytosis |

| | | |
|---|---|---|
| ARL4A | Arl4a | Phagocytosis |
| ARL4C | Arl4c | Phagocytosis |
| ARL4D | Arl4d | Phagocytosis |
| ARL5A | Arl5a | Phagocytosis |
| ARL5B | Arl5b | Phagocytosis |
| ARL5C | Arl5c | Phagocytosis |
| ARL6 | Arl6 | Phagocytosis |
| ARL8A | Arl8a | Phagocytosis |
| ARL8B | Arl8b | Phagocytosis |
| ARL9 | Arl9 | Phagocytosis |
| ARL10 | Arl10 | Phagocytosis |
| ARL11 | Arl11 | Phagocytosis |
| ARL13A | Arl13a | Phagocytosis |
| ARL13B | Arl13b | Phagocytosis |
| ARL14 | Arl14 | Phagocytosis |
| ARL15 | Arl15 | Phagocytosis |
| ARL16 | Arl16 | Phagocytosis |
| ARL17A | Arf2 | Phagocytosis |
| ARL17B | Arf2 | Phagocytosis |
| ARFRP1 | Arfrp1 | Phagocytosis |
| SAR1A | Sar1a | Phagocytosis |

| | | |
|---|---|---|
| SAR1B | Sar1b | Phagocytosis |
